# Safety and pharmacokinetics of subcutaneous administration of broadly neutralizing anti-HIV-1 monoclonal antibodies (bNAbs), given to HIV-1 exposed, uninfected neonates and infants: study protocol for a phase I trial

**DOI:** 10.1186/s12879-024-09588-3

**Published:** 2024-07-20

**Authors:** Ameena Goga, Trisha Ramraj, Logashvari Naidoo, Brodie Daniels, Masefetsane Matlou, Terusha Chetty, Reshmi Dassaye, Nobubelo K. Ngandu, Laura Galli, Tarylee Reddy, Ishen Seocharan, Qondeni Ndlangamandla, Qholokazi September, Nokwanda Ngcobo, Mayuri Reddy, Tamon Cafun-Naidoo, Kubashni Woeber, Nitesha Jeenarain, Rabia Imamdin, Keshnee Maharajh, Ashmintha Ramjeth, Thobile Bhengu, Emma Clarence, Philippe Van de Perre, Thorkild Tylleskär, Nicolas Nagot, Jean-Pierre Moles, Penny L. Moore, Nonhlanhla N. Mkhize, Lucio Gama, Stefania Dispinseri, Priscilla Biswas, Gabriella Scarlatti, the PedMAb1 clinical trial team

**Affiliations:** 1 HIV and Other Infectious Diseases Research Unit, South African Medical Research Council, Durban, South Africa; 2 Department of Paediatrics and Child Health, University of Pretoria, Pretoria, South Africa; 3 Viral Evolution and Transmission Unit, IRCCS Ospedale San Raffaele s.r.l., Milan, Italy; 4 Biostatistics Research Unit, South African Medical Research Council, Durban, South Africa; 5 Pathogenesis and Control of Chronic and Emerging Infections, University of Montpellier, INSERM, Etablissement Français du Sang; CHU Montpellier, Montpellier, France; 6 Centre for International Health, Department of Global Public Health and Primary Care, University of Bergen, Bergen, Norway; 7 SA MRC Antibody Immunity Research Unit, Faculty of Health Sciences, University of the Witwatersrand, Johannesburg, South Africa; 8 Center for HIV and STIs, National Institute for Communicable Diseases a Division of the National Health Laboratory Service, Johannesburg, South Africa; 9 Centre for the AIDS Programme of Research in South Africa (CAPRISA), Durban, South Africa; 10 Vaccine Research Center, National Institute of Allergy and Infectious Diseases, National Institute of Health, Bethesda, USA

**Keywords:** HIV, Broadly neutralizing antibody, Vertical transmission of HIV-1, Vertical transmission, Breastfeeding, Pre-exposure prophylaxis, Long-acting drugs, Safety, Infant exposed to HIV, Paediatric trial

## Abstract

**Background:**

The ambitious goal to eliminate new pediatric HIV infections by 2030 requires accelerated prevention strategies in high-risk settings such as South Africa. One approach could be pre-exposure prophylaxis (PrEP) with broadly neutralizing anti-HIV-1 monoclonal antibodies (bNAbs). The aim of our study is to define the optimal dose(s), the ideal combination(s) of bNAbs in terms of potency and breadth, and timing of subcutaneous (SC) administration(s) to prevent breast milk transmission of HIV.

**Methods:**

Two bNAbs, CAP256V2LS and VRC07-523LS, will be assessed in a sequential and randomized phase I, single-site, single-blind, dose-finding trial. We aim to investigate the 28-day safety and pharmacokinetics (PK) profile of incrementally higher doses of these bNAbs in breastfeeding HIV-1 exposed born without HIV neonates alongside standard of care antiretroviral (ARV) medication to prevent (infants) or treat (mothers) HIV infection.

The trial design includes 3 steps and 7 arms (1, 2, 3, 4, 5, 6 and 6b) with 8 infants in each arm. The first step will evaluate the safety and PK profile of the bNAbs when given alone as a single subcutaneous (SC) administration at increasing mg/kg body weight doses within 96 h of birth: arms 1, 2 and 3 at doses of 5, 10, and 20 mg/kg of CAP256V2LS, respectively; arms 4 and 5 at doses of 20 and 30 mg/kg of VRC07-523LS, respectively. Step two will evaluate the safety and PK profile of a combination of the two bNAbs administered SC at fixed doses within 96 h of birth. Step three will evaluate the safety and PK profile of the two bNAbs administered SC in combination at fixed doses, after 3 months. Arms 1 and 6 will follow sequential recruitment, whereas randomization will occur sequentially between arms (a) 2 & 4 and (b) 3 & 5. Before each randomization, a safety pause will allow review of safety data of the preceding arms.

**Discussion:**

The results of this trial will guide further studies on bNAbs to prevent breast milk transmission of HIV.

**Protocol version:**

Version 4.0 dated 15 March 2024.

**Trial registration:**

Pan African Clinical Trial Registry (PACTR): PACTR202205715278722, 21 April 2022; South African National Clinical Trial Registry (SANCTR): DOH-27–062022-6058.

**Supplementary Information:**

The online version contains supplementary material available at 10.1186/s12879-024-09588-3.

## Background and rational

The World Health Organization (WHO) recommends universal life-long antiretroviral therapy (ART) for pregnant and breastfeeding women living with HIV and short-course infant prophylaxis in HIV-1 exposed born negative newborns, and exclusive breastfeeding during the first 6 months to reduce vertical transmission of HIV-1 (MTCT) and optimize child survival (https://www.who.int/hiv/pub/mtct/programmatic_update2012/en/). The WHO criteria for MTCT elimination is now ≤ 50 (target case rate) new pediatric HIV infections per 100,000 live births (https://www.who.int/reproductivehealth/publications/emtct-hiv-syphilis/en/). While HIV-1 infections in children have decreased substantially, in 2022, approximately 130,000 (lower and upper limits 90,000 to 210,000) new infections occurred in children < 9 years; half of these occurred during breastfeeding (https://www.who.int/teams/global-hiv-hepatitis-and-stis-programmes/hiv/strategic-information/hiv-data-and-statistics). Timing of maternal HIV diagnosis, incident maternal HIV infections late in pregnancy or post-partum during breastfeeding, access to antenatal care, retention in care, early infant diagnosis and breastfeeding practices have geographic and cultural heterogeneity and strongly impact MTCT risk [1]. For example, in South Africa, where antenatal HIV prevalence has been stable at around 32% for many years, assuming that final MTCT risk is 4.3%, the pediatric case rate is 1376/100,000 live births, significantly higher than the target of 50 or fewer new pediatric HIV infections per 100,000 live births.

Postnatal transmission is high if the mother is infected during the last trimester of pregnancy or breastfeeding [1–3]). Among women achieving initial viral suppression on ART, rebound viremia occurs in up to one-third, particularly post-partum, increasing the risk of postnatal HIV-MTCT [3]. Annually approximately 6,700 babies are born at the R.K. Khan Hospital in Chatsworth, Durban where the study will be conducted. Over 2000 pregnant women are known HIV-positive and 360 to 480 additional pregnant women test HIV positive at their first antenatal visit annually and 2300 babies are born without HIV. Annually, approximately 30 babies are diagnosed with HIV and started on ART. Antenatal HIV-prevalence in this district is approximately 43%, and exclusively breastfeeding is 64% among HIV exposed infants born without HIV aged 0–14 weeks.

The ambitious goal to eliminate new pediatric HIV infections by 2030 requires accelerated prevention strategies in high-risk settings. Several approaches could be pursued. In contrast with adult individuals at high risk of HIV acquisition, the universal and equitable access to pre-exposure prophylaxis (PreP) is so far not available to children [4], despite clinical trial evidence.

Innovative biomedical solutions, including long-acting, injectable formulations, offer a paradigm shift in the pediatric HIV epidemic [5]. Modified HIV-1 bNAbs are highly potent and long-acting, and therefore have the indisputable potential for preventing MTCT perinatally and postnatally. Indeed, bNAbs for infant prophylaxis were identified as a potentially safe and durable option by the WHO-led “Paediatric Antiretroviral Drug Optimization group” and as a priority for drug development [6].

On the basis of modelling data available we propose to test the antibody directed to the CD4-binding site (CD4-bs e.g. VRC07-523) and combine it with an antibody to the V2-glycan supersite (CAP256V2). Many of the bNAbs have been engineered to enhance their therapeutic potential. Modification of the Fc-region of the bNAbs by an “LS” mutation increases binding to neonatal Fc-receptors, improving protection against lentiviral infection in non-human primates [7] and extending the bNAbs half-life to 4–6 months in adults [8]. Furthermore, isolation of more potent bNAbs (e.g. CAP256V2LS) [9] and engineering of bNAbs to increase potency (e.g. VRC07-523LS) [10] has made subcutaneous (SC) administration a realistic option in neonates and infants. Such extended half-life and elevated potency would enable protection of breastfeeding infants during the first year of life with as few as 2–3 administrations. In addition, they become particularly interesting as the volume of each administration would not exceed 1.5 ml, given the high bNAb concentration in formulations (100–150 mg/ml).

The aim of our study is to define the optimal dose(s), the ideal combination(s) of bNAbs (potency and breadth) and timing of SC administration to prevent infant HIV acquisition from birth and through breastfeeding in high-incidence countries such as South Africa.

### Trial registration

Pan African Clinical Trial Registry (PACTR): PACTR202205715278722, 27 May 2022; South African National Clinical Trial Registry: DOH-27–062022-6058, 30 June 2022.

## Methods

### Study products

In this protocol the bNAbs CAP256V2LS, never tested in pediatric populations, and VRC07-523LS were chosen based on the following characteristics:

1. epitope complementarity (CD4bs and V1V2-glycan) to target different viral regions involved in cell entry;
2. breadth for subtype C (dominant in South Africa) and other subtypes to cover also viruses circulating in other high prevalent areas beside South Africa;
3. half-life > 3–4 months in adults (VRC07-523LS), allowing large administrations intervals with the goal of providing the least number of administration during the breastfeeding period;
4. potency and availability of the formulation for SC administration, which in children is preferable to intravenous administration;
5. CAP256V2LS safety assessment in adults (CAPRISA 012B trial) showed no serious adverse events, and adverse events did not result in study product discontinuation;
6. VRC07-523LS safety assessment in the IMPAACT P1112 phase I pediatric study showed that the product is generally well tolerated, none of the severe adverse events reported were related to the study product or resulted in study product discontinuation in any participant.
7. availability through non-profit partnerships and a Target Product Profile plan to reduce costs of production.

The Vaccine Research Center (VRC), National Institute of Allergy and Infectious Diseases (NIAID), National Institutes of Health (NIH) developed VRC07-523LS, while CAP256V2LS has been developed by VRC, together with Centre for the AIDS Programme of Research in South Africa (CAPRISA). The VRC, NIAID, NIH is the sponsor of the original U.S. Food and Drug Administration (FDA) investigational new drug application (IND) to evaluate the potential clinical uses of CAP256V2LS and VRC07-523LS.

CAP256V2LS and VRC07-523LS are both recombinant human immunoglobulin G1 (IgG1) antibody produced in the Chinese Hamster Ovary (CHO) DG44 cell line in accordance with the current Good Manufacturing Practice (cGMP) regulations. The VRC Pilot Plant-VCMP is a qualified manufacturer for the release of Investigational Medicinal Products and labels clinical vials for the trial, and it manufactured, tested and released the master cell bank. Both products are approved for use in South Africa under clinical trial conditions. Neither product is subject to agreement with companies or other organizations for supply of the products.

### Study design, participants, interventions and outcomes

#### Study setting

The South African Medical Research Council (SAMRC) Chatsworth Clinical Research site (CRS) is located in the regional R.K. Khan hospital in eThekwini district, Durban, South Africa. This SAMRC CRS has the experience and infrastructure to support clinical trials with over 20 staff members including doctors, nurses, counsellors, pharmacists, data managers, quality assurance/quality control staff, laboratory staff, research assistants, community liaison officers and drivers.

#### Study design

The scheme below summarizes the 3 steps of the trial.


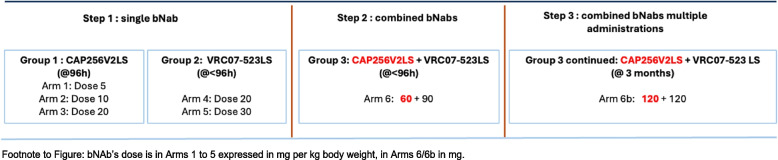


Step 1 will evaluate the safety (adverse events, AE) and pharmacokinetics (PK) profile of the bNAbs when given alone as a single SC administration at two (VRC07-523LS) or three (CAP256V2LS) increasing doses (in mg per kg body weight), respectively, within 96 h of birth, in breastfeeding exposed neonates born without HIV and receiving the standard-of-care (SOC) antiretroviral (ARV) prophylaxis. If all 5 arms of step 1 are completed with no SAEs at least possibly-related to bNAbs, the trial will proceed to step 2, involving the use of 2 bNAbs in combination (arms 6/6b). If step 1 shows no bNAb-related SAE(s), following doses of VRC07-523LS and CAP256V2LS then steps 2 and 3 will proceed, and will evaluate the highest dose that is well tolerated. The second dose (arm 6b) will be administered after 3 months, in breastfeeding exposed neonates born without HIV (Table 1). All mothers and infants will also receive SOC ARV prophylaxis.

**Table 1:** Description of each study arm

**Description of Arms**					
	**N**	**Study product**	**Dose (mg/Kg)**	**Time (from birth)**	**Recruitment**
**Arm 1:**	8	CAP256V2LS	5	< 96 h	Sequential
**Arm 2:**	8	CAP256V2LS	10	< 96 h	Randomized
**Arm 3:**	8	CAP256V2LS	20	< 96 h	Randomized
**Arm 4:**	8	VRC07-523LS	20	< 96 h	Randomized
**Arm 5**	8	VRC07-523LS	30	< 96 h	Randomized
	**N**	**Study product**	**Dose (mg)**	**Time (from birth)**	**Recruitment**
**Arm 6**	8	CAP256V2LS + VRC07-523LS	60 + 90	< 96 h	Sequential
**Arm 6b**		CAP256V2LS + VRC07-523LS	120 + 120	3 months (12 weeks)	Same infants of arm 6

Based on emerging safety data of arms 1 to 5 [11] and PK (to be published), which were adjudicated by the internal study safety committee (ISSC), independent (external) safety medical committee (ISMC) and the Data Safety and Monitoring Board (DSMB), arm 6/6b includes a fixed dose of each antibody at each time point. Furthermore, the discussions confirmed that it would be best if the second doses were administered at 12 weeks (Table 2).

**Table 2:** summarizes the step wise proceeding of the trial according to safety data

**Step 1**		Product* for dose 1	Dose 1	Timing for dose 1	Product for dose 2		Dose 2	Timing for dose 2
Arm 1:		CAP256V2LS	5 mg/kg	0- < 96 h	No second dose			
**Day 28 safety assessment and pause before the next randomization and dosing**								
Arms 2 and 4—randomised	Arm 2	CAP256V2LS	10 mg/kg					
	Arm 4	VRC07-523LS	20 mg/kg					
**Day 28 safety assessment and pause before the next randomization and dosing**								
Arms 3 and 5—randomised	Arm 3	CAP256V2LS	20 mg/kg					
	Arm 5	VRC07-523LS	30 mg/kg					
**Day 28 safety assessment and pause before the next dosing**								
**Step 2**					**Step 3 review of safety data for first dose before 2nd dose for Arm 6b:**			
Arm 6	Arm 6	CAP256V2LS + VRC07-523LS	60 mg + 90 mg	0- < 96 h	Arm 6b	CAP256V2LS + VRC07-523LS	120 mg + 120 mg	Age 3 months
**Day 28 safety assessment**								

#### Study population

Recruitment shall occur when women access antenatal clinic care from 34 weeks of gestation or post-delivery. The clinical research site has experience working with at-risk populations, given the 43% antenatal HIV prevalence in the district, and will develop a site-specific recruitment plan. Interested pregnant women will be offered an informal discussion with the site clinical research nurse or counselor. This discussion will protect the HIV status, medical information and personal circumstances of the antenatal potential participant. The potential participant or post-delivery mother will be informed about the study and will be given a copy of the Participant Information Sheet and consent form. They will have the opportunity to ask questions. Community staff will also speak with pregnant women in the community to publicize the study, and if the pregnant women are willing to attend the clinical research site post-delivery, they will be offered enrolment into the study. Mothers will only be enrolled post-delivery.

The informed consent procedure will follow Good Clinical Practice (GCP). Postnatal consent will occur for those women who have not been consented antenatally. For mothers who consent antenatally, and for mothers who consent postnatally (within 96 h), screening procedures will be undertaken. Following the receipt of screening results the mother-baby pair will be identified as eligible for enrolment or not. A checklist will be completed for all eligible mothers confirming their understanding of the study and confirming participation in the study. At this stage the mother will be provided an opportunity to withdraw from the study before the dose of study medication is administered. There is a 96-h window after delivery for women to be enrolled. This option must be balanced by the knowledge that the product is more likely to work the sooner it is administered after exposure (delivery). The timeline between randomization and study product administration will be minimized to avoid any randomized untreated infant; however, an infant will be considered enrolled in the study when he/she has received study product.

#### Inclusion and exclusion criteria

##### Maternal inclusion criteria

- Greater than or equal to 18 years of age
- Documented HIV-1 infection
- Breastfeeding at the time of consenting or willing to initiate breastfeeding in the immediate postpartum period
- Able and willing to provide a signed informed consent form to participate in the study for herself and her infant

##### Infant inclusion criteria

- Alive infant with a birth weight greater than or equal to 2.0 kg and lower than or equal to 4.0 kg
- Gestational age greater or equal to 36 weeks
- Written consent from at least one of the parents (according to South African regulations)

#### Exclusion criteria

##### Maternal exclusion criteria

- Prior participation in any HIV-1 vaccine trial
- Receipt of any other active or passive HIV immunotherapy or investigational product concurrently
- Documented or suspected serious medical illness with fetal compromise or immediate life-threatening condition (other than HIV-infection as judged by the examining clinician)
- CD4 count under 350 cells/mm^3^ within the last 6 months
- Mother not on ART
- Unable or not willing to breastfeed
- Active tuberculosis
- COVID-19 diagnosis in the past month
- Plan to relocate in 1 year
- Mother does not have her own cell phone
- Mother not able to provide two alternate contact phone numbers

##### Infant exclusion criteria

- HIV-infected on birth PCR
- Receipt of or anticipated need for blood product, immunoglobulin or immunosuppressive therapy. This includes infants who require hepatitis B immunoglobulins (HBIG) but not infants who receive hepatitis B vaccine in the new-born period
- Documented or suspected serious medical or immediate life-threatening condition
- Infant in neonatal intensive care unit (NICU) or high care requiring supplemental oxygen at time of first bNAb dose
- Known allergy to study drug or components
- Multiple birth, i.e. twins, triplets, quadruplets, etc.
- Baseline laboratory results:
  - Haemoglobin level less than 12.0 g/dL
  - Platelet count less than 100,000 cells/mm.^3^
  - Absolute neutrophil count: for infants less than 24 h old, less than 4,000 cells/mm^3^; for infants greater than 24 h old, less than 1,250 cells/mm.^3^
  - Serum glutamic pyruvic transaminase (S-GPT), alanine aminotransferase (ALT) greater than or equal to 1.25 times upper limit of age-adjusted normal
  - Serum bilirubin at a level needing phototherapy

#### Relevant concomitant care

All HIV exposed infants without HIV will receive routine SOC including prophylactic antiretroviral drugs as per national guidelines, in addition to the study product administration and will be referred to routine services for curative antiretroviral drugs in case of HIV infection.

#### Main objectives and endpoints

##### Primary objectives

1. To evaluate the safety (AE) until day 28 after a single SC administration of one bNAb, tested at increasing doses within 96 h of birth, in breastfeeding exposed neonates without HIV (arms 1 through 5).
2. To evaluate the safety (AE) until day 28 after two single SC administration of two bNAbs, tested at increasing doses within 96 h of birth, in breastfeeding exposed neonates without HIV (arm 6).
3. To evaluate the safety (AE) until day 28 after two single SC administrations of two bNAbs, within 96 h of birth and repeated at 3 months (arm 6b) or at a in breastfeeding exposed neonates without HIV.

##### Primary endpoint

The primary endpoint, applying to all the primary objectives, is the proportion of participants who develop at least one ≥ Grade 3 AE, including local and systemic reactions, lab toxicities, and/or clinical events, that is possibly, probably or definitely related to each bNAb any time from the first day of study product administration through further 28 days following bNAb(s) administration (all arms).

##### Secondary objectives

1. To evaluate the PK profile up to 6 months of each single bNAb administered, at different doses within 96 hours of birth in breastfeeding HIV exposed neonates born without HIV (arms 1 through 5).
2. To evaluate the PK profile up to 3 months of bNAbs administered in combination, at different doses within 96 hours of birth in breastfeeding exposed neonates without HIV (arm 6).
3. To evaluate the PK profile up to 6 months of each bNAb, administered in combination, in breastfeeding exposed neonates without HIV infants after a second administration at 3 months (arms 6/6b).
4. To assess safety (AE) up to 6 months after each single bNAb (arms 1 to 5), up to 3 months after the first combined bNAbs administration (arm 6) and up to 6 months after the second administration of the combined bNAbs (arm 6b).

##### Secondary endpoints

- PK curve of each single bNAb.
- Clearance, C_max_, area under the curve (AUC) and elimination half-life of each single bNAb.
- Frequency/proportion of children above the target bNAb plasma concentration (IC80) determined by in vitro studies for each bNAb.
- Frequency of participants with any type of AE including local and systemic reactions, laboratory toxicities, and/or clinical events, any time from the first day of study product administration up to 6 months after each single or combined bNabs administration.
- Frequency/proportion of participants who develop at least 1 AE including local and systemic reactions, laboratory toxicities, and/or clinical events, any time from the first day of study product administration up to 6 months after each single or after the combined second bNabs administration.
- Frequency/proportion of new HIV-1 infection among neonates/infants.

The exploratory objectives and endpoints have been omitted in this paper for brevity.

##### Interventions and study procedures

Interventions for the mother and exposed neonates without HIV are briefly summarized as follows:

Mother.

- Initial and reaffirmed informed consent
- Recent clinical history + feeding practice within 96 h of birth and at each visit till end of follow-up
- Venous blood for maternal viral load and sequencing and sensitivity to bNAbs within 96 h of birth in both blood and breast milk.

HIV exposed neonates born without HIV.

- Single bNAb administration within 96 h of birth for arms 1 through 5
- Combined bNAbs administration within 96 h of birth (arm 6) and at month 3 post-delivery (continuation of arm 6 into 6b)
- History (including AEs / SAEs + concomitant medications)
- Clinical exam (including AEs / SAEs)
- Safety assessments
- Blood for haematology (0.5 mL)
- Blood for chemistry (0.5 mL)
- Capillary blood for point of care (POC) infant HIV-1 diagnosis (up to 0.2 mL)
- Hematocrit test (1 drop)
- Randomization and enrolment within 96 h of delivery
- Dried Blood Spot (DBS) for PK (up to 0.4 mL)
- Oral fluid for PK (ELISA) obtained from 1 swab
- Venous blood for neutralising activity (0.6 mL)
- Venous blood for anti-bNAb Abs (0.6 mL)
- Breakthrough infection: in case this occurs in any arm, additional sequencing will be performed at any time point. Every infant breakthrough infection will be thoroughly investigated.

The schedule of activities planned for the mother and for the exposed infants without HIV are detailed in the following tables (Tables 3, 4 and 5). They cover a period ranging from 34 weeks of pregnancy to 1 year post-delivery. The last visit in each table corresponds to the End of Trial (EOT) visit. The amount of blood withdrawn from the exposed infants without HIV ranges from 0.5 to 3.3 mL, the latter representing the maximum at one time point (Appendix 4).

**Table 3:** Schedule of activities for the mother

	**Screening from 34 weeks pregnancy to within 96 h of delivery**	**DAYS post-delivery**	**MONTHS post-delivery**		
		**Entry visit—within 96 h of birth (Day 0)**	**2 (8 weeks)**	**3 to 6**	**7 to 12—EOT**
Time Window (± days)		3	14	14	14
Information and preparation of the potential participants	**X**				
Maternal inclusion criteria (interview, review medical history and records)	**X**				
Confirmation of mother’s HIV status (documented in medical chart or on laboratory records)	**X**				
Exclusion criteria (mother and baby)		**X**			
Informed consent process	**X**				
Recent clinical history + feeding practice on monthly basis		**X**	**X**	**X**	**X**
Venous blood for maternal viral load		X* (routine services)	**X** (only if infant is receiving a 2nd administration of bNAbs at 2 months)	**X** at 3 and 6 months (routine services)	
Venous blood for CD4 cell count (if no routine result ≤ 6 months old available)	**X**				
Milk for sequencing and sensitivity to bNAbs 5 mL up to 30 mL		**X**	**X** (only if infant is receiving a 2nd administration of bNAbs at 3 months)	**X** (only if infant is receiving a 2nd administration of bNAbs at 3 months)	
HIV sequencing in maternal blood (sensitivity to bNAb panel)		**X**	If the infant tests HIV positive a sample of breast milk will be obtained from the mother for sequencing as close as possible to that time point		
Total samples amount	5 mL blood	5–10 mL blood; 5 mL up to 30 mL breast milk	5–10 mL blood; 5 mL up to 30 mL breast milk	5–10 mL blood; 5 mL up to 30 mL breast milk	

X*: maternal viral load may be confirmed either at the screening or at the entry visit

In the following schedule of activities of Tables 4 and 5 the safety assessment consists of hematology exams: full blood count (FBC) with differential + platelets; and chemistry exams: alanine transaminase (ALT), aspartate aminotransferase (AST), total bilirubin, creatinine.

**Table 4:** Schedule of activities for HIV exposed Infants born without HIV: ARMS 1 to 5

			**DAYS post-bNAb administration**			**MONTHS post-bNAb administration**				
	**Screening (Within 96 h of birth)**	**Entry /Enrolment (within 96 h of birth)**	**3**	**14**	**28**	**2 (8 weeks)**	**3**	**4**	**5**	**6—EOT**
Time Window (± days)			+ 3	3	3	14	14	14	14	14
**bNAb administration**		**X**								
History (including AEs/SAES + concomitant medications)	**X**	**X**	**X**	**X**	**X**	**X**	**X**	**X**	**X**	**X**
Clinical exam (including AEs/SAEs)	**X**	**X**	**X**	**X**	**X**	**X**	**X**	**X**	**X**	**X**
Safety assessments		**X**	**X**	**X**	**X**	**X**	**X**	**X**	**X**	**X**

**Table 5:** Schedule of activities for HIV exposed Infants born without HIV: ARMS 6/6b

			**DAYS post – administration of 1st bNAb**						**DAYS post- administration of 2nd bNAb at 3 months (12 weeks)**				**MONTHS post- administration of 2nd bNAb at 3 months (12 weeks)**				
	**Screening (within 96 h of birth)**	**Entry/ Enrolment (within 96 h of birth)**	**1**	**3**	**14**	**28**	**56**	**84**	**1**	**3**	**14**	**28**	**2**	**3**	**4**	**5**	**6 – EOT**
Time Window (± days)			+ 1	+ 3	3	3	+ 3	+ 7	+ 1	+ 3	3	3	14	14	14	14	14
**bNabs administration**		**X**						**X**									
History (including Aes/SAEs + concomitant meds)	**X**	**X**	**X**	**X**	**X**	**X**	**X**	**X**	**X**	**X**	**X**	**X**	**X**	**X**	**X**	**X**	**X**
Clinical exam (including Aes/SAEs)	**X**	**X**	**X**	**X**	**X**	**X**	**X**	**X**	**X**	**X**	**X**	**X**	**X**	**X**	**X**	**X**	**X**
Safety assessments		**X**	**X**	**X**	**X**	**X**	**X**	**X**	**X**	**X**	**X**	**X**	**X**	**X**	**X**	**X**	**X**
Month post-delivery						**1**	**2**	**3**				**4**	**5**	**6**	**7**	**8**	**9**

### Assignment of interventions

#### Sample size calculation

A total of 48 breastfeeding HIV exposed uninfected eligible neonates will be enrolled in the study (8 neonates recruited in each arm). Power calculations were not performed for this study, given that it is a Phase 1 trial aiming to test safety and PK in a small number of participants [12]. The trial objectives specifically state an evaluation of safety that did not include comparing safety between arms; thus there was no need to perform power calculation. We have, however, carefully evaluate and presented the probability of observing at least one event under various hypothetical SAE rates and this forms the key sample size consideration. The enrolment of 48 neonates will allow the study to have 8 infants per arm (target sample size of each arm is 6) in the final safety dataset in the hypothesis of a 25% of lost to follow-up.

The probabilities reported in Table 6 highlight the likelihood of the study to detect either rare or common SAEs (Table 6). According to the reported probabilities, there is 11% chance of getting 2 SAEs in a sample of 8 participants when the true SAE proportion is 50% (i.e. common) and 5% when the true SAE proportion is 5% (i.e. rare). In addition, there is 0.4% chance of getting 0 (no) SAEs in a sample of 8 participants when the true SAE proportion is 50% (i.e. common) and 66% when the true SAE proportion is 5% (i.e. rare).

**Table 6:**
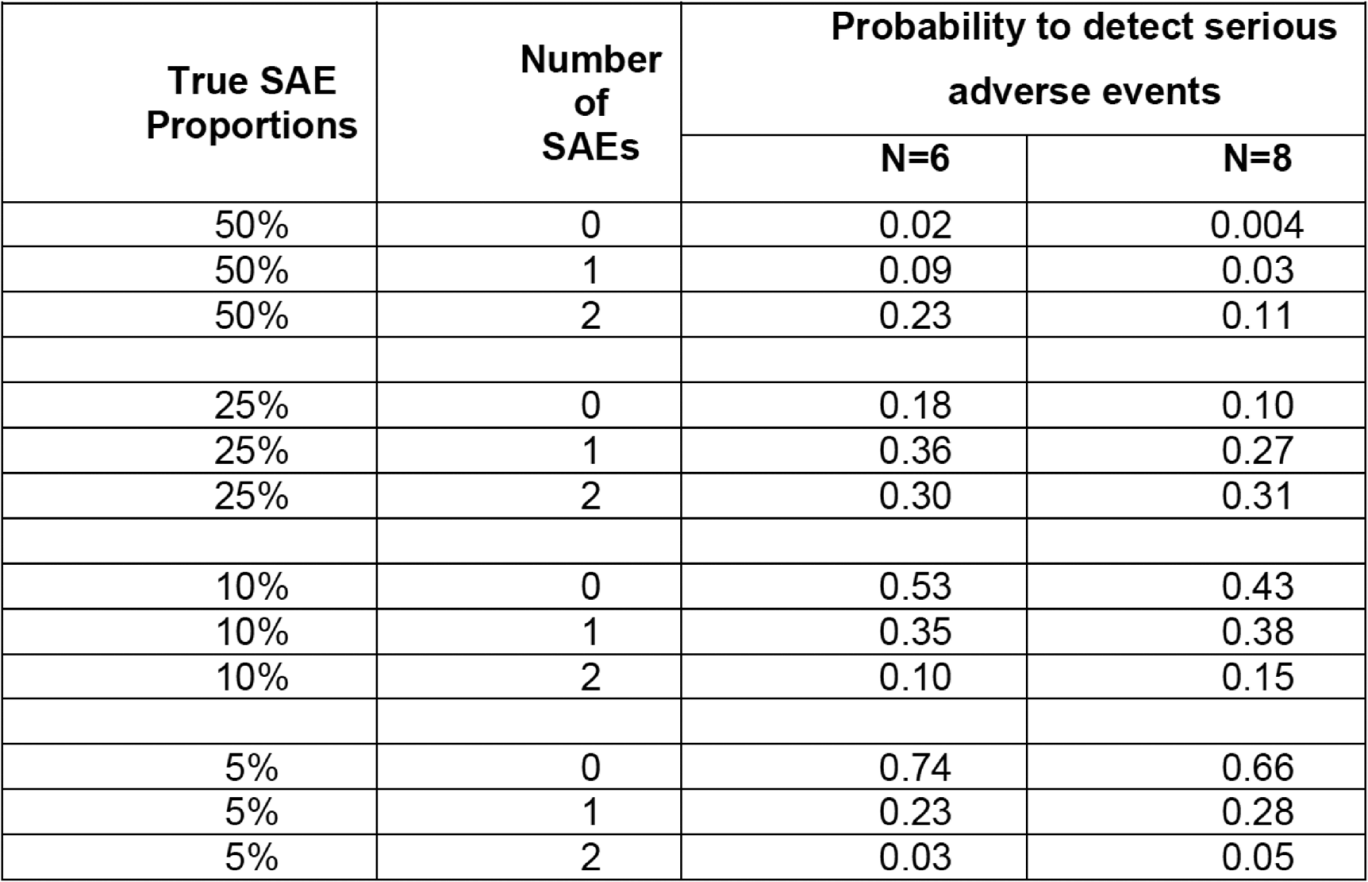
Binomial probabilities of detecting SAEs at hypothetical true event rate

The chances to pause the study are rather low since the probability to observe at least 1 SAE is at most 38%. A larger sample size would increase this probability but this would imply exposing a greater number of infants to the experimental drug.

#### Randomization and blinding

In arm 1 and 6/6b neonates exposed to HIV and born negative will be sequentially recruited, while in arms 2 or 4 and arms 3 or 5 further exposed neonates without HIV will be allocated in a ratio 1:1 using a randomization list that will be computer-generated by the trial statistician and provided through a paper-based envelope approach. The randomization list will be stratified in permuted block of size of 4; it will be used in step 1 (arms 2 to 5 only).

The trial is single-blind with neonates’ mothers blinded with respect to study product allocation to minimize the potential of contamination between arms (mothers allocated to one bNAb study product and willing to change to benefit from the other bNAb) and maximize an objective measurement of the primary outcome. We chose not to design a double-blind trial because doses within groups will be administered in a sequential way; this condition implies that even if the study staff does not know the exact dose administered, they will still be able to guess that the doses gradually administered are in increasing quantities, and, thus, eliminating at least in part the advantage of the double-blind design.

### Data collection, management and analysis

#### Data collection methods

Data will be collected for each subject in case report forms (CRFs) that will be electronic (eCRFs) and available through REDCap application version 9.3.5 (Vanderbilt University, Nashville, USA). The data will be entered directly into the REDCap server (https://samrc-redcap.mrc.ac.za) using tablets. Paper CRFs will be available as backup.

Data will be reviewed and checked for completeness and validity before, during and after data entry. The data cleaning process will include different levels of controls: (a) Source data verification and quality checks at data entry level; (b) Automatic controls on the REDCap database; (c) Database data review performed by the data manager, with the production of manual eQueries.

Entered data will be subject to visual and electronic validation by data management and monitoring staff. Any errors, inconsistencies and unexpected values in the data will be noted and queried with site staff for resolution.

Adverse events and Medical History will be coded using the Division of AIDS Table for Grading the Severity of Adult and Paediatric Adverse Events, Corrected Version 2.1, July 2017. Concomitant Medications will be coded using the World Health Organization Drug Dictionary (WHO-DDE Dictionary), the last available version.

The collected clinical data will be coded into standard medical terminology according to the International Conference on Harmonisation of Technical Requirements for Registration of Pharmaceuticals for Human Use Multidisciplinary guidelines (ICH M1).

When data review is finalised, the database will be ready for statistical analysis.

#### Data management, storage and curating data

Data management activities will be undertaken under the applicable regulatory frameworks and data management standard processes are aligned with the Good Clinical Data Management Processes (GCDMP). A governance structure will be established to oversee the development process, coordinate relevant stakeholders, facilitate decision making, advocate for resources, provide accountability for data security, and mandate the study implementation. Further, the governance structure will ensure that the study adheres to national health data policies. The SAMRC will develop and host the REDCap database. Infrastructure: Online hosting service; tablets with keyboards for data collection and linked to the central server; connectivity in facilities; centralised server/software instance model. Source documents: All the original documents for each participant in the study will be stored securely. Every effort will be made to anonymise (assigning codes and/or pseudonyms) data and protect individual confidentiality in analysis, synthesis, and dissemination activities. Original CRFs and supporting documentation regarding corrections and data changes will be digitalised. All CRFs will be assigned a unique identifier code (UIC) during data entry into the database. Mother-infant dyads will be linked using UIC. No source documents will be destroyed without permission in writing from the SAMRC. All laptops and tablets will have secure usernames and passwords. Data backup: Automated, scheduled data backups in real-time will be stored on a virtual server. We will also develop a standard operating procedure for disaster recovery planning. Given the sensitivity of mother-infant data, any backups and disaster recovery measures must be just as secure as the data in the production instance. Backups will be encrypted to the same standard as the database instance.

#### Data security and confidentiality

SAMRC security policy has been operational since 2017, ensuring standard methods and procedures are used so that security issues can be addressed expediently with minimum impact on quality. Due to the sensitivity of mother-infant data, the most secure options will be implemented: (i) SSL/https, to ensure data transmissions over the Internet are encrypted; (ii) nginx/reverse proxy, to provide another security layer between the web server and the Internet; (iii) No server-side caching, to avoid malicious manipulation of URLs. The following measures should also be put in place: (i) database encryption; (ii) hosting contractor will confirm the use of encrypted drives for the databases. The main risk to data security and confidentiality is from external users accessing personal data. To mitigate this risk, data will be pseudoanonymised before transfer and transferred using encrypted files.

#### Data sharing and access

The SAMRC has a data repository that is created on demand for each study. The repository is password-protected, and only designated individuals with the link have access to content. Data will be anonymised when stored in the repository.

##### Data sharing

Data will be shared among the Study Investigators and the study statisticians, after cleaning has been completed, in order to proceed with the analyses and results dissemination.

##### Governance of access

Anyone wishing to access the data during the study or after should write to the coordinating and co-investigators (CIs) with an outline of the analysis they would like to undertake. The CIs will then decide whether to grant access to the data to this researcher and inform the researcher within one month of the application receipt.

#### Statistical methods

The statistical analyses of the PedMAb phase I trial will be performed, unblinded as to study arm, on the “safety analysis dataset” including infants who will have received at least one dose of the randomized bNAb (alone or in combination) and who have either presented a SAE grade 3–4 during the first 28 days following first or second bNAb administration or who have completed the same follow-up with safety assessment.

The analyses will be also performed according to the per-protocol (PP) principle. The PP analysis dataset will comprise infants who have received the expected dose(s) of the randomized bNAb (alone or in combination) and who have completed all their follow-up visits (6 months) since the first or second bNAb administration.

#### Descriptive and univariate analyses

All the available safety data of each participant will be considered in the analyses; these data will be classified as severity (grade), expectedness, and potential relatedness (causality) to the study intervention. Descriptive analyses will be performed according to the study arm and the endpoints will be summarized using means with standard deviations or medians with interquartile ranges or ranges or 95% confidence intervals (CI) for quantitative variables, and proportions with their 95% CI for qualitative or categorical variables; CIs will be calculated using the exact method.

Given the small sample size of the study arms, there will be no statistical power for a formal test of differences between study arms with respect to safety. The analyses will check for clinically relevant differences in the main baseline maternal and infant characteristics between the following study arms: 2 vs 3, 4 vs 5, 6 vs 3 and 5; the presence of relevant imbalances in these characteristics among arms will be eventually tested by calculating differences [between means (standardised effect size) or proportions] with the corresponding 95% confidence intervals.

#### Primary analysis on primary endpoint

The primary endpoint of each step is the proportion of neonates who develop at least one ≥ 1 Grade 3 adverse events (AE) possibly, probably or definitely related to each bNAb that occurred within 28 days after a first or a second dose of bNAbs administration (alone or in combination). The primary endpoint will be described according to the study arm, using proportions with the corresponding 95% CI.

#### Secondary analyses on secondary endpoints

AEs rates, expressed as number of events divided by number of person-days of follow-up and calculated by univariate Poisson regression, will be also estimated among enrolled neonates.

Pharmacokinetic parameters will be estimated by a non-compartmental analysis (NCA) performed using WinNonlin® version 8.0 (Certara, St. Louis, MO, USA) applied on data collected from neonates who completed the study. The primary PK endpoints will be: clearance, maximum plasma concentration (C_max_) and area under the concentration–time curve (AUC) from time zero to the last quantifiable time point (AUClast). C_max_ values will be compared with those at baseline (pre-administration of bNAbs) using the Wilcoxon signed-rank test. Bioequivalence between study arms will be assessed using the geometric mean ratio (GMR) and 90% confidence interval (90% CI) of AUClast and C_max_. The conventional bioequivalence criterion of 0.80 will be used.

The other endpoints of the trials will be summarized with descriptive analyses; they will be described according to the study arm and, if assessable, by month of follow-up until a maximum of 6 months since first or second bNAbs administration, using means with standard deviations or medians with interquartile ranges or ranges or 95%CI for quantitative variables, and proportions with their 95% CI for qualitative or categorical variables. Non-parametric tests that will be considered to test changes in continuous measures during follow-up are: Wilcoxon signed-rank test or Friedman test.

### Monitoring

#### Safety monitoring

The 2015 South African operational guidelines on Ministerial consent for non-therapeutic health research with minors will be followed. We will categorise this study as non-therapeutic research because this is a phase I dose-finding, safety and PK study; we do not know whether the interventions, at the doses and intervals tested, will have therapeutic benefit. The following conditions will be applied:

- Condition 1: All consent will be provided by one or both parent/guardian who is the main person looking after the child. Consent procedures will follow GCP procedures, i.e. information sheets will be translated in vernacular language and routine care will not be affected by non-participation.
- Condition 2: The research may carry a risk as CAP256V2LS has not been tested in an infant population; however, CAP256V2LS is an antibody that was specifically derived from a South African adult patient; it specifically targets the strain of HIV pervasive in southern Africa and is highly potent. The trial with CAP256V2LS is initiated with available adult safety data of CAPRISA 012B [13]. Additionally, children will be closely monitored to ensure safety and document reactogenicity and adverse events.

Babies will be observed for a minimum of 2 up to 4 h after the first antibody dose and for at least an hour after the second antibody dose and will be reviewed face-to-face at days 1, 3, 14 and 28 post-delivery, then monthly up to month 6 post-delivery for babies receiving a single administration and up to months 3 for babies receiving the first bNAb administration and for additional 6 months post second bNAb administration for babies receiving two administrations. Additionally, study doctors and nurses will be available telephonically and in-person 24/7, to receive any calls or to see mothers and their babies face-to-face, in between these scheduled visit time points.

The first antibody dose will be given in a hospital setting (either within the RK Khan hospital or at the clinical research site which is located on the hospital precinct) to ensure close proximity to intensive care unit facilities and access to more than one paediatrician and nurses.

Furthermore, the Chatsworth CRS has community liaison staff, which would also be available in the community, directly or through the community advisory board to provide any support to mothers and their babies and to facilitate their access to immediate health care.

Lastly, through an existing memorandum of understanding with the R.K. Khan Hospital, any baby with any AE needing hospital-level care will be referred timeously. This will be facilitated by the fact that the Chatsworth CRS is on the premises of the R.K. Khan Hospital and within walking distance to the pediatric outpatient clinic and wards. Additionally, for this study, a research pediatrician will be employed on a full-time basis to complement existing staff and ensure the welfare of mothers and babies. This study pediatrician will oversee the safety of children and will work with existing clinicians at the site.

#### Toxicity management and enrolment in study arms

Arm 1 will follow a sequential recruitment, and all the other arms will be randomized. Simultaneous recruitment will occur for arms 2 and 4; arms 3 and 5; and will proceed as follows:

When the planned 8 infants of arm 1 have been enrolled, given study product and completed 28 days of follow-up, the study investigators will review interim safety data; there will be a 1-month pause in the enrolment and administration of subsequent doses (arms 2 and 4) during this review. If review of safety data reveals Grade 3 or higher AE possibly, probably, or definitely related to product administration, the study investigators will decide to consult with the ISMC. If review of safety data reveals no SAE, enrolment into subsequent dose arms will be opened. The same work-flow will apply to arm 2 and 4, arms 3 and 5, and arm 6. Infants enrolled in arm 6 will be treated with a second administration of bNAbs after 3 months constituting arm 6b; these babies will be followed according to the criteria described in the following paragraph.

#### Toxicity management second administration – Study arm 6b

A second bNAbs administration will occur in arm 6b and should be provided according to the following criteria:

- In case of Grade 3 toxicities after the first bNAbs administration (regardless of their relationship to study product), the second dose should not be administered until after consultation with the study investigators AND improvement of the event to < Grade 3. After approval by the study investigators and the ISMC, the second administration may occur. If the event persists, no further administrations may occur.
- In case of Grade 4 toxicities considered as not related or possibly or probably not related, the second administration may occur after improvement to < Grade 3 and consultation with the study investigators and the ISMC. If the event persists, no further administrations may occur.

Subsequent administration should not occur

- In case of any Grade 4 toxicities considered as possibly, probably, or definitely related to study product,.
- In case the infant has documented or suspected serious medical or immediate life-threatening condition at the time of the planned second bNAb administration.
- In case the infant is admitted in ICU or high care requiring supplemental oxygen at time of planned second bNAbs administration.
- In case an infant is determined to have acquired HIV after the first bNAb administration or
- In the case of an infant who has stopped breastfeeding (before the second dose).

#### Adverse events and serious adverse events

The mothers of exposed neonates born negative will be given and trained on using a standardised diary card to note any reactions at the injection site(s) and/or any AEs/SAEs that may occur after the bNAbs administration/s. Each AE and SAE that occurs during the study must be documented in the paper medical records (chart notes) of the patient in accordance with the standard clinical practice of the study investigators and on the AEs/SAE page of the e-CRF. A separate set of SAE pages must be used for each SAE.

The study investigators should try to formulate a diagnosis of the event on the basis of signs, symptoms, and/or other clinical information and record it on the AE and/or SAE pages. Similarly, with clinically significant abnormal laboratory values or other clinical examinations are consistent with the definition of an AE or SAE. It is important that the study investigators provide his/her judgment regarding the severity, expectedness, and potential relationship (causality) of the event to the study therapy on the initial SAE form.

#### Safety committees

The Internal Study Safety Committee (ISSC) will review a safety report every 2 weeks, and meet monthly, or ad hoc when the need arises. The ISSC will include the three pediatricians on the study team, the site PI, the Sub-Investigator, the study managers and the Clinical Research Organization (CRO) representative.

An Independent Safety Medical Committee (ISMC) will meet every 6-months to review the safety data or ad hoc if there is a safety trigger. The ISMC is formed by 3 pediatricians and a neonatologist.

The Data and Safety Monitoring Board (DSMB) will review safety, PK and other data and will meet every 6-months or ad hoc if there is a specific need. The DSMB is an interdisciplinary group formed by 5 members (1 statistician, 1 virologist, 1 pharmacologist, 1 sociologist, 1 pediatrician).

Members of the ISMC and DSMB will be independent of study conduct and free of conflict of interest, and measures will be in place to minimize perceived conflict of interest.

The ISMC and DSMB will assess progress, safety endpoints and recommend whether the study should continue, be modified or halted.

#### Trial monitoring

An external CRO will be hired to conduct monitoring of all aspects of the trial as per GCP.

#### Analyses and stopping rules

Stopping rules have been defined and will be applied in order to protect the study participants from unnecessary exposure to the monoclonal antibody, should the safety profile prove unacceptable in this population. Unblinded interim exploratory (as this trial is proof-of-concept, no flexible alpha spending function approach will be employed) analyses are planned when the last infant of each study arm will have completed 28 days of follow-up since the first dose of bNAb(s) (individually administered or in combination). These analyses may lead to stop enrolment of subsequent dose arms, adjustment of doses or of the second bNAbs administration.

If at any time during the study, the following triggers are met:

i) any of the infants dies or has a life-threatening adverse event or any Grade 4 event that is possibly, probably or definitely related to CAP256V2LS or VRC07-523LS administration;
ii) two or more of the eight infants in each arm of the study have a Grade ≥ 3 adverse event at least possibly related to CAP256V2LS or VRC07-523LS administration (excluding Grade 3 neutropenia and anemia, and hyperbilirubinemia), the study investigators will pause the study for evaluation and will request that a DSMB review be performed within 7 days since the event occurrence. The DSMB will decide what additional data they would like to see and advise the study investigators on how to proceed. DSMB may decide on premature termination of some dose/arms (not of the trial); the decision may apply to either the CAP256V2LS or the VRC07-523LS arms (to each single dose/arm) and considers the occurrence in the CAP256V2LS arms independent of that in the VRC07-523LS arms. If the DSMB decides that further enrolment or administration cannot be continued, the study will stop accrual or administration but continue safety follow-up of all enrolled infants.

#### Missing data/loss to follow-up

All reasonable efforts will be taken to minimise loss to follow-up, which is expected to be minimal as data collection for primary and secondary endpoints. Contact details recording and tracking systems are in place to ensure a low rate of lost in the study; in addition, in the sample size calculation, we allowed for a 25% of loss to follow‐up. The number and percentage of participants with complete follow-up information at day 28 and at 6 months, since first or second dose of bNAbs administration (alone or in combination), will be reported.

#### Roles and responsibilities

Coordinating Investigators

- Ameena Goga (AG)
  - HIV and other Infectious Diseases Research Unit
  - South African Medical Research Council (SAMRC)
  - 1 Soutpansberg Road
  - Arcadia, Private bag X 385001
  - Pretoria, South Africa (SA)
  - Phone: +27 12 3398524
  - E-mail: ameena.goga@mrc.ac.za
- Gabriella Scarlatti (GS)
  - Viral Evolution and Transmission Unit
  - Ospedale San Raffaele srl. (OSR)
  - Via Olgettina 60
  - 20132 Milan, Italy
  - Phone: +39 02 2643 4906
  - E-mail: scarlatti.gabriella@hsr.it

Co-investigators

- Philippe Van de Perre, INSERM/Univ Montpellier/EFS, France
- Penny Moore, Wits Health Consortium (Pty) Ltd (WHC), SA
- Thorkild Tylleskär (TT), Universitetet i Bergen, Norway

Overall project management

- Stefania Dispinseri (SD), OSR, Italy
- Trisha Ramraj (TR), SAMRC, SA
- Kubashni Woeber, SAMRC, SA (Central Laboratory coordination in SA)

Statistician and Data Management

- Tarylee Reddy, SAMRC, SA (Chief statistician)
- Ishen Seocharan, SAMRC, SA (Data manager)

Clinical Team at Chatsworth Clinical Research Site (CRS)

- Logashvari Naidoo (LN), SA (Principal Investigator)
- Nitesha Jeenarain, SA (Site CRS leader)
- Mayuri Reddy and Tamon Cafun-Naidoo, SA (Pharmacists)
- Emma Clarence (Pediatrician and sub-investigator)

Researchers

- Terusha Chetty, SAMRC, SA
- Reshmi Dassaye, SAMRC, SA
- Brodie Daniels, SAMRC, SA
- Nobubelo Ngandu, SAMRC, SA
- Carol Crowther, WHC, SA
- Jean-Pierre Moles, INSERM/Univ Montpellier/EFS, France
- Nicolas Nagot, INSERM/Univ Montpellier/EFS, France
- Yoann Cazaubon, Univ Montpellier, France

This trial is sponsored by the South African Medical Research Council (SAMRC), PO Box 19070, Tygerberg, 7505, Francie van Zijl Drive, Parow Valley, Cape Town, Telephone: 021 938 0911, www.samrc.ac.za.

Study sponsor and funders do not have any role in study design; collection, management, analysis, and interpretation of data; writing of the report; and the decision to submit the report for publication.

## Ethics and dissemination

A rigorous ethical and regulatory approval process specified by the national and institutional regulation will be followed. SAHPRA (South African Health Products Regulatory Authority) notification of approval is required before participants can be enrolled in this study.

As per South African Good Clinical Practice Guidelines (SA-GCPs), the G-EthicsHR, and the G-GPHlthCare, the informed consent form (ICF) and patient information sheet(s) are essential documents that will be reviewed and approved by the SAMRC Human Research Ethics Committee (HREC), an accredited ethics committee (EC) based in South Africa and provided to SAHPRA with the clinical trial application.

Protocol amendments will follow the same procedure. The study will be implemented after consultation and buy-in from the Chatsworth CRS Community Working Group (CWG). These discussions commenced in 2020, and updates will continue throughout study implementation.

### Consent or assent

Written informed consent for study participation will be obtained before any study related procedures are performed. Mothers will be consented and screened prior to delivery or shortly after delivery of their infants, but randomization of the mother-infant pair will not proceed until the infant eligibility criteria have been confirmed. According to the SA-GCPs, the ICF and any patient information sheet(s) should be written in English and in the vernacular language that the participant is able to understand. Thus, we will provide information to the participants in a language that the participant understands and in a manner that takes into account the participant’s level of literacy, understanding, values, and personal belief systems. The Coordinator or a person designated by the PI will provide research study information to the participant and/or his/her legal representative(s), or guardian(s). The ICF content will be briefly and clearly presented, without coercion or unduly influencing a potential participant to enroll in the clinical trial. The original signed informed consent documents (ICF) will be retained by the investigator and a copy will be given to the participant for his/her record.

The current v4.0 dated 15 March 2024 Protocol, with its ICF and PIS, has been approved by SAHPRA and by the SAMRC HREC on Apr 23, 2024 (Appendices 2 and 3). Relevant changes to previous protocol versions are listed in Appendices 1.

The study has also been notified and approved by the Institutional Review Board of the Montpellier University Hospital, Montpellier, France on March 3, 2022; by the Ethics Committee of the San Raffaele Hospital, Milan, Italy on May 11, 2022; and by the Regional Committee for Medical and Healthcare Research Ethics (REK) north, Norway on December 12, 2022.

### Dissemination policy

The study team will disseminate the trial results by sharing the results with the CWG, with the scientific community at national and international conferences, through peer-reviewed journal publications, and by sharing results with policy makers nationally, and with the World Health Organization.

### Supplementary Information

Supplementary Material 1: Appendix 1. Table 1. Changes to the protocol.

Supplementary Material 2: Appendix 2. PedMAb1_IC_Version 4.0 Dated 15Mar2024_ENGLISH.

Supplementary Material 3: Appendix 3. PedMAb1_PIS_Version 4.0 Dated 15Mar2024_ENGLISH.

Supplementary Material 4: Appendix 4.word Table 7: Schedule of sample collection for HIV exposed infants without HIV: ARMS 1 to 5. And Table 8: Schedule of sample collection for HIV exposed infants without HIV: ARMS 6/6b. Schedule for collection of the biological samples of arms 1-5 and arms 6/6b.

## Data Availability

No datasets were generated or analysed during the current study.

## Abbreviations

ADA: Anti-bNAb antibody
AE: Adverse events
ALT: Alanine aminotransferase
ART: Antiretroviral therapy
ARV: Antiretroviral (prophylaxis)
AST: Aspartate aminotransferase
AUC: Area under the curve
bNAbs: Broadly neutralizing (anti-HIV-1 monoclonal) antibodies
CAP256V2LS: A broadly neutralising anti-HIV-1 monoclonal
CAPRISA: Centre for the AIDS Programme of Research in South Africa
CD4: A receptor (or a T-cell lymphocyte with many such receptors)
cGMP: Current good manufacturing practice
CHO: Chinese Hamster Ovary DG44 cell line
CI: Confidence intervals
C_max_: Maximum plasma concentration
CRF: Case report form
CRO: Clinical research organisation
CRS: Chatsworth Clinical Research site
DBS: Dried blood spots
DSMB: Data safety and monitoring board/committee
e-CRF: Electronic case report form
ELISA: Enzyme-linked immunosorbent assay
EOT: End-of-trial visit
FBC: Full blood count
FDA: U.S. Food and Drug Administration
GCDMP: Good clinical data management processes
GCP: Good clinical practice
GMR: Geometric mean ratio
GPT: Glutamic pyruvic transaminase
HBIG: Hepatitis B immunoglobulin
HIV-1: Human immunodeficiency virus type 1
IgG1: Recombinant human immunoglobulin
IND: Investigational new drug application
ISMC: Independent safety medical committee
ISSC: Internal study safety committee
MTCT: Mother-to-child-transmission of HIV-1
NCA: Non-compartmental analysis
NIAID: National Institute of Allergy and Infectious Diseases
NICU: Neonatal intensive care unit
NIH: National Institutes of Health
PCR: Polymerase chain reaction
PI: Principal investigator
PK: Pharmacokinetics
PMTCT: Prevention of mother-to-child-transmission of HIV-1
POC: Point-of-care (test)
PrEP: Pre-exposure prophylaxis
REDCap: Research Electronic Data Capture
SAE: Serious adverse event
SAMRC: South Africa Medical Research Council
SAHPRA: South African Health Products Regulatory Authority
SC: Subcutaneous injection
SOA: Schedule of activities
UIC: Unique identifier code
VRC07-523LS: A broadly neutralising anti-HIV-1 monoclonal
V1V2-glycan: HIV envelope variable loop 1 and 2 glycan
VRC: Vaccine Research Center
WHO: World Health Organization

## References

[1] Drake AL, Wagner A, Richardson B, John-Stewart G. Incident HIV during Pregnancy and Postpartum and Risk of Mother-to-Child HIV Transmission: A Systematic Review and Meta-Analysis. PLoS Med. 2014;11(2):e1001608. 24586123 10.1371/journal.pmed.1001608 PMC3934828

[2] Dinh TH, Delaney KP, Goga A, Jackson D, Lombard C, Woldesenbet S, et al. Impact of Maternal HIV Seroconversion during Pregnancy on Early Mother to Child Transmission of HIV (MTCT) Measured at 4–8 Weeks Postpartum in South Africa 2011–2012: A National Population-Based Evaluation. PLoS ONE. 2015;10(5):e0125525. 25942423 10.1371/journal.pone.0125525 PMC4420458

[3] Myer L, Dunning L, Lesosky M, Hsiao NY, Phillips T, Petro G, et al. Frequency of viremic episodes in HIV-infected women initiating antiretroviral therapy during pregnancy: a cohort study. Clin Infect Dis. 2017;64(4):422–7. 27927852 10.1093/cid/ciw792 PMC5849096

[4] Van de Perre P, Kankasa C, Nagot N, Meda N, Tumwine JK, Coutsoudis A, et al. Pre-exposure prophylaxis for infants exposed to HIV through breast feeding. BMJ. 2017;9:j1053. 10.1136/bmj.j1053 28279960

[5] Van de Perre P, Goga A, Ngandu N, Nagot N, Moodley D, King R, et al. Eliminating postnatal HIV transmission in high incidence areas: need for complementary biomedical interventions. Lancet. 2021;397(10281):1316–24. 33812490 10.1016/S0140-6736(21)00570-5

[6] Penazzato M, Townsend CL, Rakhmanina N, Cheng Y, Archary M, Cressey TR, et al. Prioritising the most needed paediatric antiretroviral formulations: the PADO4 list. Lancet HIV. 2019;6(9):e623–31. 31498110 10.1016/S2352-3018(19)30193-6

[7] Ko SY, Pegu A, Rudicell RS, Yang Z yong, Joyce MG, Chen X, et al. Enhanced neonatal Fc receptor function improves protection against primate SHIV infection. Nature. 2014;514(7524):642–5. 25119033 10.1038/nature13612 PMC4433741

[8] Gaudinski MR, Coates EE, Houser KV, Chen GL, Yamshchikov G, Saunders JG, et al. Safety and pharmacokinetics of the Fc-modified HIV-1 human monoclonal antibody VRC01LS: A Phase 1 open-label clinical trial in healthy adults. PLoS Med. 2018;15(1):e1002493. 29364886 10.1371/journal.pmed.1002493 PMC5783347

[9] Doria-Rose NA, Bhiman JN, Roark RS, Schramm CA, Gorman J, Chuang GY, et al. New Member of the V1V2-Directed CAP256-VRC26 Lineage That Shows Increased Breadth and Exceptional Potency. J Virol. 2016;90(1):76–91. 26468542 10.1128/JVI.01791-15 PMC4702551

[10] Rudicell RS, Kwon Y Do, Ko SY, Pegu A, Louder MK, Georgiev IS, et al. Enhanced potency of a broadly neutralizing HIV-1 antibody in vitro improves protection against lentiviral infection in vivo. J Virol. 2014;88(21):12669–82. 10.1128/JVI.02213-14 PMC4248941 25142607

[11] Goga A, Ramraj T, Naidoo L, Matlou M, Daniels B, Chetty T, et al. PedMAb1 clinical trial: Safety assessment of CAP256V2LS to prevent breastmilk HIV transmission in HIV-1 exposed uninfected neonates. 11th EDCTP Forum. Paris: 7-10 November. BMJ Glob Health. 2023;8:A16–7.

[12] Cook TD, DeMets DL, editors. Introduction to Statistical Methods for Clinical Trials. London: Chapman & Hall; 2008. ISBN 9781584880271.

[13] Mahomed S, Garrett N, Capparelli EV, Osman F, Mkhize NN, Harkoo I, et al. Safety and pharmacokinetics of escalating doses of neutralising monoclonal antibody CAP256V2LS administered with and without VRC07-523LS in HIV-negative women in South Africa (CAPRISA 012B): a phase 1, dose-escalation, randomised controlled trial. Lancet HIV. 2023;10(4):e230–43. 37001964 10.1016/S2352-3018(23)00003-6

