# Supplementary material for: Safety and pharmacokinetics of subcutaneous administration of broadly neutralizing anti-HIV-1 monoclonal antibodies (bNAbs), given to HIV-1 exposed, uninfected neonates and infants: study protocol for a phase I trial
Source: BMC Infect Dis. 2024 Jul 20;24:712. doi: 10.1186/s12879-024-09588-3 (PMC11264722; doi:10.1186/s12879-024-09588-3)
Supplement: Supplementary file 1 — Supplementary Material 1: Appendix 1. Table 1. Changes to the protocol. [file 12879_2024_9588_MOESM1_ESM.docx]

**Appendix 1**

Table 1. Changes to the protocol

| **v2 01 April 2022** | **v3 24 November 2022** | **v4 15 March 2024** |
| --- | --- | --- |
| Exclusion criteria: added “mother not on ART” | The 72h window for enrollment has been widened to 96h  to accommodate logistic and organizational needs to improve the recruitment rate without affecting infection risk and safety of participants. | Target sample size changed from 72 to 48 neonates because arm 7, 8 and 9 has been removed from the sudy |
| Venous blood for CD4 cell  count (if no routine result ≤6  months old available) was added in mother’s activity table at screening | The recruitment site has been widened to delivery facilities in the catchment area of the RK Khan hospital to improve the recruitment rate and to offer study participation to mothers living in the surrounding area who do not attend antenatal clinic at RK Khan hospital. | Time for second administration has been set at 3 months instead of 2 months and  or at a time point between 3 and 6 months post-delivery |
| Number of study visits for arm 7 and 8 change from 14 to 14-17 depending on the time of second dose injection | The first bNAb dose for the 3rd to 8th infant in each arm might be administered at the Chatsworth Research Site (CRS), which is located on the premises of the RK Khan hospital. | Doses for arm 6 and 6b changed from mg/kg to fixed dose, as follow:  First administration:  CAP256 + VRC07  10+30 mg/kg  To  60 + 90 mg  Second administration:  CAP256 + VRC07  10 + 20 mg/kg  To  120 + 120 mg |
| PIS and IC changed according to the new version of the protocol | Standard-of-care ART for mothers and Infants might have changed as revisions of the national PMTCT protocol which wasunderway in 2022 | Randomization has occurs sequentially for arm 2-4 and arm 3-5. Arm 6 will be enrolled after safety assessment of the previous arms. |
|  | The amount of blood withdrawn from the HEU infants has changed from range 0.6 to 3.6 mL, to range 0.5-3.3 ml the latter representing the maximum at one time point. | Updated results of CAPRISA 012B and VRC 611 studies were reported |
|  | Concerning Relatedness, text has been added to provide background information on relatedness to study product and added definition on Events not related to study product | Table 1 “Study scheme”, table 3 “overview of study scheme” and table 4 “description of study arms” have changed to comply with removal of arms 7, 8 and 9. |
|  | An independent safety medical committee (ISMC) has been introduced to assess progress | Table 8a “Schedule of activity for HEU infants: arms 6/6b”:  Day 1 post bNAbs administration has been introduced for sample collection;  Day 84 has become the day of the second administration instead of day 56 |
|  | Breastmilk volume has changed from  20 to 30 ml to 5 up to 30 ml | In table 8b “Schedule of sample collection for HEU Infants: ARMS 6/6b” sample collection has changed according to table 8a |
|  | Amount of blood to be spotted on DBS has changed from 80ul to 70ul | The statistical software has changed from SAS version 9.4 to STATA or R. |
|  | 0.5mL EDTA blood would be used for HIV diagnosis POC test (0.1mL), and a repeat test if needed (total 0.2mL). The remaining blood would be used to spot the cards with 70μL for each spot | Ballard score has not been used to asses gestational age |
|  | Neutralization activity had to be measured at 3 months and not at 2 months | PIS and IC changed according to the new version of the protocol |
|  | PIS and IC changed according to the new version of the protocol |  |
