# Supplementary material for: Safety and pharmacokinetics of subcutaneous administration of broadly neutralizing anti-HIV-1 monoclonal antibodies (bNAbs), given to HIV-1 exposed, uninfected neonates and infants: study protocol for a phase I trial
Source: BMC Infect Dis. 2024 Jul 20;24:712. doi: 10.1186/s12879-024-09588-3 (PMC11264722; doi:10.1186/s12879-024-09588-3)
Supplement: Supplementary file 2 — Supplementary Material 2: Appendix 2. PedMAb1_IC_Version 4.0 Dated 15Mar2024_ENGLISH. [file 12879_2024_9588_MOESM2_ESM.pdf]

# Phase I study to determine Safety and Pharmacokinetics of subcutaneous administration of potent and broadly neutralizing anti-HIV-1 monoclonal antibodies (bNAbs), given to HIV-1 exposed uninfected (HEU) neonates and infants.

## PedMAb1 - INFORMED CONSENT FORM

**Principal Investigators:**
Dr. Ameena Goga - South African Medical Research Council (SAMRC)
Dr. Logashvari Naidoo - Chatsworth clinical research site PI (SAMRC)
Dr. Gabriella Scarlatti - Ospedale San Raffaele, srl

## INTRODUCTION

Dear Mrs, Miss

You are being invited to participate in a research study entitled:

"Phase I study to determine Safety and Pharmacokinetics of subcutaneous administration of potent and broad anti-HIV-1 neutralizing monoclonal antibodies (bNAbs), given to HIV-1 exposed uninfected (HEU) neonates and infants". In short we call it PedMAb1.

Please read this consent form or ask someone to read it to you. If you decide to join the study, we will ask you to sign or make your mark on this form. We will offer you a copy to keep. We will ask you questions to see if we have explained everything clearly. You can also ask us questions about the study.

Research is not the same as treatment or medical care. The purpose of a research study is to answer scientific questions.

This study is sponsored by the South African Medical Research Council (SAMRC). It is funded by an organisation called the European and Developing Countries Clinical Trial Partnership, or EDCTP.

## 1. WHY ARE WE DOING THIS STUDY?

Babies can get infected with human immunodeficiency virus (HIV) during pregnancy, delivery or breastfeeding. Breastfeeding is very important to protect babies from diarrhoea, pneumonia and other sicknesses. However, some babies born to mothers living with HIV who have high viral load may get HIV during breastfeeding. We are looking for better ways to stop babies getting infected with HIV during breastfeeding. Even though we give mothers and babies

medicines called ARVs to prevent HIV infection to the baby, sometimes we forget to take the ARVs. Sometimes babies vomit or spit out the ARVs. Our study is about finding a better way, like an immunisation to stop babies from getting HIV during breastfeeding. While you and your baby are on your routine medicines, we are going to test the safety and proper dosing of two study products or medicines called HIV-1, broadly neutralizing monoclonal antibodies (bNAbs). These will work like an immunisation. We will only enrol your baby into the study if he / she has a negative HIV test at delivery. If your baby tests HIV positive at birth we will make sure he / she gets the right medicine. We will not enrol him / her into this study.

You and your baby are being asked to take part in this research study because

1. Your baby is exposed to HIV and
2. you are willing to breastfeed for at least 4 to 6 months after your baby is born.

**2. SPECIFIC BACKGROUND**

During this study, we will provide study products or medicine called HIV-1, broadly neutralizing monoclonal antibodies (bNAbs). These will work like an immunisation.

bNAbs are relatively new. They have been tested in adults and are safe. They are very strong and protect against HIV infection. They last for a long time in the blood. This means that they do not have to be given every day – this is different from ARVs which need to be used every day. There are 2 bNAbs that will be used in this study, named VRC07-523LS and CAP256V2LS. We will refer to them as VRC07 and CAP256. These may be used alone (one at a time) or in combination. Compared to a single administration of each single bNAb the combination of the 2 bNAbs may provide a broader and stronger protection against HIV transmission during breastfeeding.

VRC07 and CAP256 have been shown to attach to and inactivate many HIV viruses in laboratory experiments. *In vivo* studies in several animal models, including monkeys showed that they protected baby monkeys from getting HIV. In humans, VRC07 is currently being studied or investigated in 15 clinical trials or studies. One study is being done in babies exposed to HIV. CAP256 is being investigated in 2 studies, none are in infants. These studies show that the 2 bNAbs are safe in adults. VRC07 has been tested in babies and there have been no safety concerns. CAP256 has not been tested in babies / children yet. However there are no safety

concerns in adults. The combination of CAP 256 and VRC07 has not been tested in babies/children yet. The two antibodies can be used individually or in combination in prevention studies in babies, to evaluate the safety, proper dosing, and administration routes. This is a proof-of-concept study to test the feasibility of sub-cutaneous (SC) administration of bNAbs to HIV-exposed uninfected neonates, but the study products are not necessarily the ones that will proceed in their development.

The 2 bNAbs will be given (or administered) either alone or together (in combination) at increasing doses. The timing of the administration is within 96h from birth and/or at 12 weeks (3 months) after delivery. The main objective of this study is to evaluate the safety of the bNAbs administration at each dose, combination and timing. The total amount of blood drawn from your baby at any visit will not be higher than 5 mL (1 teaspoon). Your baby will receive the routine ARV medicine he / she needs. If your baby tests HIV positive at birth we will make sure he / she gets the right medicine. We will not enrol him / her into this study. If your baby tests HIV negative at birth he will be randomly allocated to one of the groups of the study. The groups are called arms. Details of the different arms in which your baby could be randomly allocated are described further in this Informed Consent.

## 3. WHAT DO I HAVE TO DO TO BE IN THE STUDY?

If you decide that you want to be in the study, we will check to make sure you can enrol in this study. The screening can start after 34 weeks of pregnancy.

In the following sections we will refer to volumes of blood or breast milk and, to give an idea, 1 teaspoon corresponds to 5 mL.

### 3.1 Screening visit to see if you can be in the study

- Screening checklist followed by informed consent process.
- We will ask for your permission to review your medical records to see if you have been diagnosed with HIV.
- We will also review the records for confirmation of your HIV status, viral load and for other illnesses that you may have had and that might affect your baby's health.
- We will ask you questions about other studies you might have been in. We will also ask about health conditions and treatments you might have received. If your baby is expected to

receive any immunoglobulin products after birth, then you and your baby cannot be part of this study.

- The review of your medical history and the interview with you may be done while you are still pregnant and shortly after you deliver your baby. If it is done while you are still pregnant, you will also be asked more questions after your baby's birth.

**3.2 Entry Visit**

- If we have not already done so, we will ask for your permission to review your medical records to see if you have been diagnosed with HIV according to the study criteria.
- We will also review the records for other illnesses you may have had, and that might affect your baby's health.
- We will ask you questions about other studies you might have been in and health conditions and treatments you might have received.
- <u>Intended specimen draw from mother:</u> Within 96 hours after delivery, we will collect a 5-10 mL (1-2 teaspoons) sample of your blood and about 5 mL up to 30 mL (1-6 teaspoons) of your breast milk. Some of this blood and breast milk will be used to check if you have HIV (if it is not clear in your medical record), how much HIV is in your blood or breast milk and what type of HIV-1 is in your breast milk (if it is present).
- <u>Intended specimen draw from baby:</u> Within 96 hours of delivery we will collect approximately 1 mL blood (one fifth of a teaspoon) from your baby to check for anaemia, and to check liver and kidney function. We will also draw blood to test your baby for HIV. If your baby's HIV test comes back as positive we will make sure your baby gets care including antiretroviral treatment. You and your baby will not be able to continue on this study if he/she tests HIV positive, but we will make sure you are supported and get the care you need.
- Please note that the screening and entry visit may be combined on the same day.
- If you and your baby are eligible to enrol in the study then your baby's visits and specimen draws are detailed in **Section 4**.

### 3.3 Monthly Visits after delivery

- All babies enrolled in this study will receive the study product within 96h of birth. Some babies will receive a second administration of the study product at 12 weeks (3 months) post-delivery.
- You will need to take the baby to the Chatsworth Clinical Research Site (CRS) to check for adverse events and other safety tests at day 1, 3 and 14 after each study product administration and every month up to 6 months or up to 9 months, depending on which study arm your baby is enrolled in.

### 3.4 Visit at week 12 weeks (3 months) after delivery

This visit is planned if your baby has been allocated to a study arm that will receive a second administration of the study product at 12 weeks (3 months) since birth.
We will collect a 5-10 mL sample of your blood and about 5 mL up to 30 mL of your breast milk for the purposes described at the entry visit.

## 4. WHAT WILL HAPPEN TO MY BABY IF HE/SHE IS IN THIS STUDY?

If you allow your baby to be in this study, he/she will undergo a screening visit. This aims to check whether your baby meets the inclusion criteria. If yes, you and your baby will need to attend several visits. The number of visits may vary from 9 to 16, covering approximately 6 months up to 9 months after birth. This depends on whether your baby receives one or two administrations of the study product. The visits will be scheduled with the study staff.
Below is a brief description of the different arms of this study. Your baby will be allocated to one group in the study. Based on this group allocation your baby may receive one or two SC administrations of bNAbs.

### 4.1 Study arms:

Your baby will be allocated to one of 6 study arms. You cannot choose which study arm you and your baby are allocated to. This will depend on the stage of the study and will occur randomly (by chance).

**<u>Arm 1:</u>** baby will receive within 96h of birth CAP256 at a dose of 5 mg/Kg of weight

**<u>Arm 2 or 4:</u>** baby will be randomly allocated to arm 2 or 4:

- arm 2: CAP256 at 10 mg/Kg of weight within 96h of birth;
- arm 4: VRC07 at 20 mg/ Kg of weight within 96h of birth;

**<u>Arm 3 or 5:</u>** baby will be randomly allocated to arm 3 or 5:

- arm 3: CAP256 at 20 mg/Kg of weight within 96h of birth;
- arm 5: VRC07 at 30 mg/ Kg of weight within 96h of birth;

**<u>Arm 6:</u>** baby will be allocated to arm 6:

- arm 6: CAP256 at fixed dose of 60mg + VRC07 at fixed dose of 90mg within 96h of birth;

The same babies of arm 6 will receive a 2$^{nd}$ study product administration:

- arm 6b: CAP256 at fixed dose of 120mg + VRC07 at fixed dose of 120mg at 12 weeks;

The number of injections your baby receives will depend on your baby's weight and age for the first five arms:

**Arms 1-5:** Babies will only receive an injection at birth. They will receive 1 injection except if they fall under Arm 5 and are the maximum weight of 4kg. The 4kg baby will need 1.2mL which will be given as 2 injections.

**Arm 6 and 6b:** All babies will receive 2 - 3 injections at birth, one of 0.6 and 1-2 of 0.9 mL. At 3 months all babies will receive 2 injections of 1.2mL of volume. We will try and give as few injections as possible and safe.

### 4.2 Study visits for your baby

- If your baby is in any arm from 1 to 5 there will be **9** study visits:
  - within 96h of birth and at days 3, 14, 28 and then at months 2, 3, 4, 5, 6 since birth.
- If your baby will be in arm 6 → 6b there will be **16** study visits:
  - within 96h of birth, at days 1, 3, 14, 28, Day 56 (month 2) and then at months 3 (second administration), day 1, 3, day 14, day 28 after second administration and at months 5, 6, 7, 8, 9 since birth.

<u>**All study arms will have the following procedures:**</u>

**4.3 Screening visit to see if your baby can be in the study**

If you agree to allow your baby to be in this study, we will do some tests to make sure your baby is able to enter this study. This visit may take place in the hospital or at our research site within 96 hours of birth. This visit will last about 1 hour.

At this visit, we will

- Ask if you are willing for your baby to complete all scheduled study visits if he/she is enrolled in the study.
- Ask about your baby's medical history. This will include how your baby has been doing, if he/she has been sick, conditions he/she may have, and any medicines he/she has been taking. This will also include your permission to review your baby's medical records to check for medical and laboratory conditions and any treatment your baby may have received.
- Collect some demographic data such as race, ethnicity and gender.
- Do a physical examination. This may include length, weight, head measurement and vital signs such as temperature, blood pressure, heart rate, and respiratory rate (how fast the baby breathes).
- Draw blood from either a vein (called a venipuncture) or from your baby's heel (called a heel stick) to test your baby for HIV. This will only be done if it has not already been done as part of your baby's standard of care (the care your baby receives regardless of the study).
- <u>If your baby results are HIV-positive, then he/she will not enter the study.</u>
- If your baby is confirmed to be HIV negative, we will draw blood (1/5 of a teaspoon: 1 mL)
    - to know how well your baby's liver and kidneys are working (chemistry)
    - to know the status of your baby's red blood cells and immune cells (hematology)
- <u>The screening visit may occur at the same time as the entry visit</u>.

**4.4 Entry visit (within 96h of birth) for all study arms**

If your baby results are HIV-negative, then he/she is able to be in the study and will receive the first product administration.

- Your baby will be given the product through an SC administration in the skin of the upper leg. Your baby will be observed for about 4 hours to check the injection site with assessments at 15, 30 and 60 minutes post injection and make sure that he/she is well.
- We will draw 0.4 mL of blood (less than 1/5 of a teaspoon) to measure a baseline level of the study product to compare results at your baby's next visit. This will be drawn before your baby receives the SC product administration.
- We will also collect some saliva to check if and how much study product may be contained in it. The saliva is collected by placing a small pad (about the size of a thumbnail attached to the stick) between the gum and cheek of your baby's mouth. After a minute or two, we will remove the pad and put it in a container. The pad will be tested to measure the amount of study product in your baby's saliva.

- We will draw blood (just under half a teaspoon: 2 mL):
  a) to measure in vitro whether there is an HIV inhibitory activity given by the study product
  b) to check the reactivity of your baby's immune cells
- You will be asked to note any reactions at the injection site(s), adverse events and other information about your baby's health on a diary card.

**4.5 Day 3 and day 14 visit for Arms 1-5 after the first bNAb administration and Day 1, 3 and 14 post 1st and 2nd bNAb administration for Arms 6 and 6b**

These visits will last about 1 hour and we will:

1) review the diary card
2) ask how is your breastfeeding proceeding
3) ask safety assessment questions about the study product administration
4) check the injection site
5) do a physical examination of your baby
6) draw a tiny amount of blood to make an HIV diagnosis (this will not be done at the Day 1 visit after the first or 2nd bNAb administration for Arms 6 and 6b)
7) draw 0.5 mL (less than 1/5 of a teaspoon) of blood to measure how much study product is present in the blood

8) collect a small amount of saliva to check if and how much study product it contains

Note: At the Day 1 visit only a physical examination of your baby will be documented and a draw of 1.0 mL of blood to measure how much study product is present in the blood.

**4.6 Day 28 visit after first bNAb administration for all study arms**

- In addition to actions 2) through 8) enlisted in day 3/14 visits
- We will draw blood (1/5 of a teaspoon: 1 mL)
  - to know how well your baby's liver and kidneys are working (chemistry)
  - to know the status of your baby's immune cells (hematology)
- We will draw blood (just under half a teaspoon: 2mL):
  a) to measure in vitro whether there is an HIV inhibitory activity given by the study product
  b) to check the reactivity of your baby's immune cells

**4.7 Month 2 (or 8 weeks) visit for all study arms**

- This visit will last about 1 hour, and we will go through actions 2) to 8) enlisted above in the days 3/14 visits.

**<u>Study arms 1-5 will have the following procedures</u>**

**4.8 Month 3 visit for study arms 1-5**

This visit will last about 1 hour, and we will go through actions 2) to 8) enlisted above in the days 3/14 visits.

- We will draw blood (just under half a teaspoon: 2mL):
  a) to measure *in vitro* whether there is an HIV inhibitory activity given by the study product
  b) to check the reactivity of your baby's immune cells

**4.9 Month 4 visit for study arms 1-5**

This visit will last about 1 hour, and we will go through actions 2) to 8) enlisted above in the days 3/14 visits.

- We will draw blood (1/5 of a teaspoon: 1mL):

a) to measure *in vitro* whether there is an HIV inhibitory activity given by the study product

**4.10 Month 5 visit for study arms 1-5**

This visit will last about 1 hour, and we will go through actions 2) to 8) enlisted above in the days 3/14 visits.

**4.11 Month 6 visit for study arms 1-5**

This visit will last about 1 hour, and we will go through actions 2) to 8) enlisted above in the days 3/14 visits.

We will draw blood (just under half a teaspoon: 2mL):

a) to measure *in vitro* whether there is an HIV inhibitory activity given by the study product
b) to check the reactivity of your baby's immune cells

<u>This is the last study visit programmed for all babies enrolled in arms 1 through 5.</u>

**<u>Study arms 6/6b will have the following procedures:</u>**

**These study arms will follow the same procedures as above for the screening and entry study visit, Day 1, 3, 14 28 and 56 post-study product administration (see sections 4.3, 4.4, 4.5,4.6 and 4.7). For month 3 (12-weeks) (arm 6b) the additional study procedures are listed below. The study arm 6b will follow the same procedures as above for Day 1, 3 and 14, 28 post-second study product administration.**

**4.12 Month 3 (or 12 weeks) visit (second product administration)**

- As stated above, this visit will last about 3 hour, and we will go through actions 2) to 8) enlisted above in the days 3/14 visits.
- <u>Because the baby has been assigned to continue in arm 6b, then the following will also occur:</u>
- He/she will then receive a second administration of each of the two study products (one SC injection in each thigh). You and your baby will need to stay one extra hour for the observation time after the product administration.
- We will draw blood (1/5 of a teaspoon: 1 mL)
  - to know how well your baby's liver and kidneys are working (chemistry)
  - to know the status of your baby's red blood cells and immune cells (hematology)
- We will draw blood (just under half a teaspoon: 2mL):

a) to measure in vitro whether there is an HIV inhibitory activity given by the study product
b) to check the reactivity of your baby's immune cells

**4.13 Month 4 visit for arm 6b (same as Day 28 post second bNAb administration)**

This visit will last about 1 hour, and we will go through actions 1) to 8) enlisted above in the days 3/14 visits.

- We will draw blood (1/5 of a teaspoon: 1 mL)
  - to know how well your baby's liver and kidneys are working (chemistry)
  - to know the status of your baby's red blood cells and immune cells (hematology)
- We will draw blood (1/5 of a teaspoon: 1mL):
  a) to measure in vitro whether there is an HIV inhibitory activity given by the study product
- We will assess safety after the second bNAbs dose

**4.14 Month 5 visit for arm 6b**

This visit will last about 1 hour, and we will go through actions 2) to 8) enlisted above in the days 3/14 visits.

**4.15 Month 6 visit for study arm 6b**

This visit will last about 1 hour, and we will go through actions 2) to 8) enlisted above in the days 3/14 visits.

- We will also draw blood (1/5 of a teaspoon: 1mL):
  a) to measure *in vitro* whether there is an HIV inhibitory activity given by the study product

**4.16 Month 7 visit for study arm 6b**

This visit will last about 1 hour, and we will go through actions 2) to 8) enlisted above in the days 3/14 visits.

- We will draw blood (just under half a teaspoon: 2mL):
  a) to measure *in vitro* whether there is an HIV inhibitory activity given by the study product
  b) to check the reactivity of your baby's immune cells

**4.17 Month 8 visit for arm 6b**

This visit will last about 1 hour, and we will go through actions 2) to 8) enlisted above in the days 3/14 visits.

**4.18 Month 9 visit for arm 6b**

This visit will last about 1 hour, and we will go through actions 2) to 8) enlisted above in the days 3/14 visits.

- We will draw blood (just under half a teaspoon: 2mL):

a) to measure *in vitro* whether there is an HIV inhibitory activity given by the study product

b) to check the reactivity of your baby's immune cells to the study product.

<u>This is the last study visit programmed for all babies enrolled in arm 6/6b.</u>

**<u>5.1 Early Discontinuation visit for any study arm</u>**

If your baby cannot continue with the study after receiving the study product administration due to a Serious Adverse Event (SAE) related to it, we will ask you to return for a final Early Discontinuation visit that will last about 1 hour. At this visit, we will:

- record the SAE appropriately and notify the regulatory agencies
- review your baby's medical history
- do a physical examination of your baby

**5.2 Results of Tests**

Throughout the study, you will be informed of the results of the HIV testing and routine blood tests (chemistry and hematology) when they become available. The results of the other blood tests and the results of the saliva testing will be used for research purposes only. These results will not be reported to you or your doctor.

**6. HOW MANY PEOPLE WILL TAKE PART IN THIS STUDY?**

This study will include 48 mothers and their babies. Multiple pregnancy (twins, triplets, quadruplets) are not eligible for this study.

**7. HOW LONG WILL I/MY CHILD/BABY BE IN THIS STUDY?**

You will only be in the study for as long as it takes to screen you and determine that you are eligible for the study (about one day) and have your blood and breast milk collected at the visits planned (see point 3). Your baby will be in this study for 6 or 9 months, depending on which study arm your baby will be enrolled in (see point 4).

**8. WHY WOULD THE DOCTOR TAKE MY BABY OFF THIS STUDY EARLY?**

The study doctor may need to take you/your baby off the study early without your permission if:

- your infant does not receive the study product
- you are unable to return for infant follow-up evaluations
- you are unable to comply with the study requirements which may seriously interfere with whether the study results will be correct
- the investigator determines that further participation would be detrimental to you or your baby's health or well-being.

**9. WHAT IF MY BABY IS FOUND TO BE HIV-INFECTED?**

As noted in the visit details, your baby will be tested for HIV at each study visit, except the Day 1 visit after the first and second bNAb administration for Arms 6 and 6b.

If your baby has a positive HIV test at any time after the first study product administration, the second administration will be cancelled (if your baby was enrolled in arms 6).

However, your baby will still be followed up.

It will be important that your baby returns to the clinic as soon as possible for a test to confirm whether he / she is infected with HIV. About 5 mL (almost 1 teaspoon) of blood will be drawn to look for how much HIV is in your baby's blood and to measure your baby's T cells. If this visit is not a regular study visit day, we will ask about medical history since the last visit, do a physical examination, and draw an additional 1 mL (1/5 of a teaspoon) to look at the amount of study product in your baby's blood. If you consent, an additional 1 mL of blood will be drawn for storage (see section 18 below).

You and your baby will return about 2 weeks later when confirmatory test results are available. These results will be shared with you and with your baby's healthcare provider. If your baby is

confirmed to be HIV-infected, we will refer you to the ARV clinic to get treatment for your baby. We will ask you to return at 2 weeks, 4 weeks, and monthly until your child's doctor begins ART for your child. Whenever possible, these will be scheduled to coincide with ARV visits. At each study visit, the following will be done:

- History
- Physical examination
- Blood collection for HIV virus level
- Blood collection for study product level
- If you consent, an additional 1 mL of blood will be drawn for storage.

Once your baby is started on anti-HIV medications, your baby's study visits can be according to the standard of care at the ARV clinic. Your baby will continue to return for study visits as regularly scheduled. At each of these visits, we will check that your baby is well. If your baby is having blood drawn as part of the standard of care, and if you consent, we will ask the nurse or doctor to draw an additional 1 mL (1/5 of a teaspoon) of blood for storage (see section 18 below).

## 10. WHAT ARE THE RISKS OF THE STUDY?

This section describes the risks we know about. There may also be unknown risks, even serious ones that we do not know about. We will tell you if we learn anything new that may affect you/your baby's health and/or willingness to stay in the study.

*Risks of routine medical procedures:* In this study, we will do some routine medical procedures, including taking blood and collecting saliva. Blood collection can cause bruising, pain and infrequently fainting or lightheadedness. The saliva pad may be slightly uncomfortable for a few minutes while in the baby's mouth.

*Risks from subcutaneous (SC) injections:* It is possible that your baby may have some side effects from the study product injection. General risks of an injection method include stinging, discomfort, pain, soreness, redness, bruising, swelling or a tiny cut at the needle insertion site and, rarely, local infection.

*Risks of the study products:* It is impossible for the antibody to give your infant HIV. However, we do not know if the antibody will decrease, increase, or not change the chance of your infant

from becoming infected with HIV if he/she is exposed to the virus via breastfeeding. All current information tells us that it decreases the chance.

In humans, VRC07-523LS is currently investigated in 15 clinical trials including one in HIV-1-exposed infants. To date VRC07-523LS is being given to babies in Cape Town, and 26 adults have received VRC07-523LS. More than 40 infants have received the very similar VRC01 and VRC01LS bNAbs. CAP256 is currently investigated in 2 studies, none in infants. So far, no serious side effects have been seen in the babies who have received VRC01 or VRC01LS by subcutaneous injection. There has been some bruising, swelling and redness at the injection site, but these get better quickly. Most side effects tend to occur within the first 24 hours. Your baby will be observed for at least four hours after he/she has received the first dose of VRC07-523LS or CAP256V2LS. If your baby receives the second dose of each product, he/she will be observed for at least one hour after that dose.

Side effects are expected to be rare but may include fever, chills, shaking, nausea, vomiting, pain, headache, dizziness, trouble breathing, high or low blood pressure, itchiness, rash, hives, lip or face swelling, diarrhea, racing heart or chest pain. These reactions may be related to how fast the antibody product is given. The product will be administered to your baby slowly.

Monoclonal antibody products have a small risk of serious allergic reactions, including anaphylaxis. Anaphylaxis may include difficulty breathing, low blood pressure, hives or rash, swelling in the mouth and face. These reactions are life-threatening. Such reactions usually occur quickly after the injection. Therefore, your baby will be observed in the clinic for at least four hours after the first dose and at least one hour after the second dose (if received). The clinics are prepared to treat allergic reactions.

Serum sickness is a delayed-type of allergic reaction that may occur a week or two after a product is given. This reaction may include hives or rash, fever, big lymph nodes (glands in the neck and along the body), and pains in the joints. You will be contacted after the visit to make sure your baby is not having this type of reaction.

## 11. INCIDENTAL FINDINGS

The objective of this study is to help prevent the transmission of HIV from a mother to her child. During this clinical trial, however, it is possible that we make findings that may have implications for your well-being or that of your family and your child, other than the knowledge

and management of an HIV infection. Such findings are called "Incidental Findings": findings concerning an individual research participant that has potential health or reproductive importance and is discovered in the course of conducting research but is beyond the aims of the study.

What are the benefits, risks, and implications of receiving Incidental Findings?

There is no guarantee that you will get any benefit from being informed of any incidental finding but, in some cases, receiving this information could allow you to seek clinical care, adopt preventive practices, advise family members, and/or make informed healthcare decisions.

However, there are potential risks and inconveniences to receiving information about incidental findings. One is the risk of distress from learning the finding. Even if therapy, treatment and/or counselling is available to address the finding, it may not work. In addition, there is always a risk that the finding is later determined to be inaccurate or false. There may also be risks from receiving the finding that are unknown at this time. For example, sometimes there may be implications for your ability to find a job or you may face stigma in your community. Any of these outcomes could cause you and your family emotional and psychological distress, as well as financial hardship.

In order to reduce the risk of sharing findings that are inaccurate, your health worker will first try to confirm the validity of those findings by running confirmatory tests or assessments before discussing them with you.

Decision to receive Incidental Findings:

Whether or not you choose to receive the finding is voluntary. You will not lose any benefits or rights you would normally have if you choose not to receive the finding.

Even if you do not want to receive incidental findings at this time, you may contact your study caretaker in the future if you change your mind and would like to consent to receiving any incidental findings in the future. Either way, your decision will not affect your care or your participation in this study.

You are encouraged to discuss the option of receiving the incidental finding with your family and outside sources as well as your medical providers (such as physicians, counsellors, healthcare worker, and medical specialist).

**12. ARE THERE BENEFITS TO TAKING PART IN THIS STUDY?**

If you and your baby take part in this study, it is possible that the study product/s may prevent the transmission of HIV to the baby from the mother in a better way than ARV alone. It is also possible that your baby may receive no benefit from being in this study. Information learned from this study may help others who are exposed to or infected with HIV.

**13. WHAT OTHER CHOICES DOES MY BABY HAVE BESIDES THIS STUDY?**

You and your baby do not have to participate in this study. You may choose to have the standard of care**.** You and your baby will still receive care without being in the study. All babies will receive standard antiretroviral medications after their birth to decrease the likelihood that they may become HIV-infected whether on the study or not.

**14. WHAT ABOUT CONFIDENTIALITY?**

We will take several steps to protect you and your baby's personal information. Although the risk is very low, it is possible that you or your baby's personal information could be given to someone who should not have it. If that happened, you could face discrimination, stress, and embarrassment. We can tell you more about how we will protect your personal information if you would like it.

**15. WHAT ARE THE COSTS TO ME?**

There is no cost to you for the study visits, examinations, blood tests or the study product administrations your baby will be given.

You or your medical aid or the routine government health services will be responsible for costs that are considered standard of care. This refers to care you or your baby would receive whether or not you or your baby are on the study. We will write on your baby's Road to Health Booklet (RTHB) that your baby is part of this study, and fill in the RTHB every time the baby comes for a follow-up visit, to ensure continuity of care for your baby.

**16. REIMBURSEMENT?**

You will not be paid for your baby to be in this study. You will be reimbursed for the cost of your time, inconvenience and expenses, on the days of your study visit, in accordance with the

South African Health Products Regulatory Authority (SAHPRA) guidelines. The amount for a full study visit will be R450.00.

**17. WHAT HAPPENS IF I AM/MY CHILD/BABY IS INJURED?**

If your baby is injured as a result of being in this study, your baby will be given immediate treatment for his/her injuries. The cost for this treatment will be charged to the study insurance company. You will not be giving up any of your legal rights by signing this consent form. This policy will follow the guidelines for payment of study-related illness or injury approved by the Association of the British Pharmaceutical Industry ("ABPI Guidelines"). You can get a copy of these ABPI Guidelines from us if you wish. The South African Medical Research Council (HIV and other Infectious Diseases Research Unit) will facilitate your additional treatment for your research related injuries through the clinical trial insurance for this study (Name of Insurance Company: Marsh).

**18. WHAT ARE MY/MY BABY'S RIGHTS AS A RESEARCH PARTICIPANT?**

Taking part in this study is completely voluntary. You may choose not to participate in this study, or not to allow your baby to participate in this study. You may leave this study or take your baby out of the study at any time. You and your baby will be treated the same no matter what you decide.

We will tell you about new information from this or other studies that may affect you/your baby's health, welfare or willingness to stay in this study. The community engagement team will disseminate the results to the study participants at the end of the study

**19. STORAGE OF BLOOD SAMPLES**

Some of your and your baby's blood and saliva will be stored (with protectors of identity) and used for future HIV-related research. Your baby can still participate in this study even if you decide that you do not want to have his/her blood stored for later testing.

Your/your baby's samples will be stored at the SAMRC or our partner laboratories. Only approved researchers will have access to them. People who work at the facility will also have access to your/your child's samples to keep track of them. These people won't have information that directly identifies you/your child. Your/your baby's samples will not be sold or directly

used to produce commercial products. All proposed research studies using your/your baby's samples will be reviewed by the SAMRC and the HREC (Human Research Ethics Committee) in South Africa. There is no time limit on how long your/your baby's samples will be stored.

The researchers do not plan to contact you or your baby's regular doctor with the results of studies done using your/your baby's stored samples. This is because research studies are often done with experimental procedures. The results of such studies should not be used to make decisions about your/your child's medical care. If the researchers decide that the result of a certain study provides important information for your/your baby's medical care, your/your baby's study doctor will be notified. If you would like to be contacted with this sort of information, you must notify the study staff of any changes in your address or phone number.

You may decide that you do not want your/your baby's samples stored for future research studies. You/your baby can still participate in this study even if you make this decision.

You may withdraw your consent for the storage and use of your/your baby's samples at any time. If you withdraw your consent, these stored samples will be destroyed.

There are two types of testing that can be done with the specimens that are in storage. Please read the following statements carefully and then mark your initials and date in the appropriate space provided.

### 19.1. Testing for General HIV-related studies

Researchers would like to store your/your baby's specimens to understand how HIV causes disease and complications, and how best to treat or prevent HIV infection and its complications. They need specimens from people who have HIV and from those who do not. Sometimes, too, the specimens can be used to learn something about new problems that people with HIV have like liver disease, diabetes, and heart disease. These general studies would not include any genetic testing (looking at what makes up you/your child's cells).

Benefits: There are no direct benefits to you/your baby. You/your baby will be helping researchers learn more about how to help people with HIV or at risk of HIV infection.

Risks: The specimens would be collected as part of your/your baby's study visits. Blood collection can cause bruising, pain and infrequently fainting or lightheadedness. Once in

storage, there are few risks. Your/your baby's name will not be available to the staff at the laboratory or to the scientists who may be doing any future test.

### 19.2. Testing for Special HIV-related studies

Researchers in this study would also like to store your/your baby's specimens to understand how HIV causes disease and complications, and how best to treat or prevent HIV infection and its complications through looking at how each person's genetic makeup (your/your baby's DNA or unique cell makeup) either protects them or puts them at greater risk. It may be that researchers use some of your/your baby's blood to make a "cell line." That means the blood cells can keep dividing and give an endless supply of your/your baby's DNA for tests to be done in the future. This kind of information will be particularly important as scientists work toward a vaccine that could protect people from AIDS. They need specimens from people who have HIV and from those who do not.

Benefits: There are no direct benefits to you/your baby. You/your baby will be helping researchers learn more about how to help people with HIV or at risk of HIV infection.

Risks: The specimens would be collected as part of your/your baby's study visits. Blood collection can cause bruising, pain and infrequently fainting or lightheadedness. Once in storage, there are few risks. Your/your baby's name will not be available to the laboratory staff or to the scientists who may be doing any future test. Since there are no plans to give participants the results of the tests performed on their stored specimens, you will not receive any information on your/your baby's genetic makeup.

## 20. WHAT DO I DO IF I HAVE QUESTIONS OR PROBLEMS?

If you have questions about this study you should first discuss them with the study Team or the related Ethics Committee.

Tel: (012) 501 0410 for the study Team; or

Prof. Ameena Goga: Ameena.Goga@mrc.ac.za

Dr. Logashvari Naidoo: (031) 242 3688 or Logashvari.Naidoo@mrc.ac.za

If you have any additional queries, please contact the SAMRC Human Research Ethics Committee (HREC): Adri Labuschagne, tel. (021) 938 0687; adri.labuschagne@mrc.ac.za

The study has been structured in accordance with the Declaration of Helsinki (last updated October 2013) which deals with the recommendations guiding doctors in biomedical research involving human participants, the Ethics in Health Research: Principles, Structures and Processes Second Edition 2015, and Guidelines for Good Practice in the Conduct of Clinical Trials in Human Participants in South Africa. We can provide you with copies of these guidelines if you wish to review them.

If you have not been provided with answers to your satisfaction, you should write to the South African Health Products Regulatory Authority (SAHPRA) who provides regulatory approval for the study at:

The Chief Executive Officer
South African Health Products Regulatory Authority
Loftus Park
Building A
402 Kirkness Street
Arcadia, Pretoria
0083

E-mail: Boitumelo.Semete@sahpra.org.za
Tel: 012 501 0413

# CONSENT TO PARTICIPATE IN PHASE I STUDY TO DETERMINE SAFETY AND PHARMACOKINETICS OF SUBCUTANEOUS ADMINISTRATION OF POTENT AND BROADLY NEUTRALIZING ANTI-HIV-1 MONOCLONAL ANTIBODIES (BNABS), GIVEN TO HIV-1 EXPOSED UNINFECTED (HEU) NEONATES AND INFANTS.

## If you agree to join the study, please read the following and then sign below.

- I have read and understood this information.
- This study has been explained to me.
- All my questions about the study, the bNAbs (study product) and possible risks and benefits have been answered to my satisfaction.
- I give permission for my personal information and health to be collected from medical and laboratory records as well as other approved data sources and kept in the Sponsor's database and understand that any data shared and used for the study as explained in this consent form will be Coded Data (anonymized).
- I understand that my personal and anonymised information ('data') may be shared with the Sponsor, designated Monitors, Auditors and Inspectors as per study protocol and Good Clinical Practice.
- I freely agree to participate in this research study as described and understand that I am free to withdraw at any time during the study.
- I understand that I will be given a signed copy of this document to keep.

In accordance with the provisions of the Protection of Personal Information Act 4 of 2013 (as amended), I hereby consent:

a. To my personal information (hereinafter 'data') being collected, processed, shared and stored in accordance with the research protocol as approved by the South African Medical Research Council's Human Research Ethics Committee (SAMRC HREC);
b. To my anonymised data being shared, processed and transferred by third parties and between third parties, and where relevant beyond the jurisdictional borders of South Africa;
c. To all findings and results flowing from my anonymised data being broadly shared and published on the conclusion of the research.

I have been informed that the study doctor/staff may inform my regular doctors (if any) about my participation in this study, and I agree to this. (You may still be in this study even if you do not agree to this.)

| Yes | No | Not applicable, I have no other doctors |
| --- | --- | --- |
| | | |

I agree to allow my blood/breastmilk samples and my baby's blood and saliva samples to be stored for use in future approved general HIV-related research studies.

| Yes | No |
| --- | --- |
| | |

I agree to allow my blood/breastmilk samples and my baby's blood and saliva samples to be stored for use in future -approved, special HIV-related research studies.

| Yes | No |
| --- | --- |
| | |

I agree to receive Incidental Findings if encountered during the course of this clinical trial.

| Yes | No |
| --- | --- |
| | |

Printed name and surname of participant in full

Signature of participant Date (dd/mmm/yyyy)

**For participants who are unable to read or write, a witness should complete the signature block below:**

________________________________________
Printed name and surname of witness in full

______________________________________________________________
Signature of witness Date (dd/mmm/yyyy)

__________________________________________________________________
Printed name and surname of person obtaining consent

__________________________________________________________________
Signature of person obtaining consent Date (dd/mmm/yyyy)
