# Supplementary material for: Safety and pharmacokinetics of subcutaneous administration of broadly neutralizing anti-HIV-1 monoclonal antibodies (bNAbs), given to HIV-1 exposed, uninfected neonates and infants: study protocol for a phase I trial
Source: BMC Infect Dis. 2024 Jul 20;24:712. doi: 10.1186/s12879-024-09588-3 (PMC11264722; doi:10.1186/s12879-024-09588-3)
Supplement: Supplementary file 3 — Supplementary Material 3: Appendix 3. PedMAb1_PIS_Version 4.0 Dated 15Mar2024_ENGLISH. [file 12879_2024_9588_MOESM3_ESM.pdf]

PedMAb phase I study to determine Safety and Pharmacokinetics of subcutaneous administration of potent and broadly neutralizing anti-HIV-1 monoclonal antibodies (bNAbs), given to HIV-1 exposed uninfected (HEU) neonates and infants.

PATIENT INFORMATION SHEET

PedMAb

Sponsor: South African Medical Research Council (SAMRC),
Francie van Zijl Drive, Parowvallei, Cape Town; 7505 Tygerberg, South Africa

You are invited to take part in this **PedMAb1** research study. We are trying to find a way to eliminate (get rid of) HIV transmission from mother to baby after delivery. Please take some time to read the full informed consent form. **The information presented here summarises the study. Please ask study staff any questions** about this study that you do not fully understand. It is very important that you agree with the study and that you fully understand what this research study is about and how you could be involved. Your participation is **entirely voluntary** and you are free to decline to participate**. If you say no, this will not affect you negatively by any means or in any form.** You are also **free to withdraw from the study at any point** in time, even if you agree to take part in it at the beginning.

This study has been approved by the **Committee for Human Research at the South African Medical Research Council** and will be conducted according to the ethical guidelines and principles of the international Declaration of Helsinki, international and local Guidelines for Good Clinical Practice and Ethical Guidelines for Research.

Background Information

<u>What is HIV-1?</u>

The human immunodeficiency virus (HIV-1) is a retrovirus that infects cells of the human immune system, destroying or affecting their function. In the early stages of infection infected individuals do not present with symptoms, however, as the infection progresses, the immune system becomes weaker, and the infected individuals become more susceptible to so-called opportunistic infections. The most advanced stage of HIV-1 infection is acquired immunodeficiency syndrome (AIDS). It can take 10-15 years for an HIV-infected person to develop AIDS; antiretroviral drugs can slow down the process even further.

HIV is transmitted through unprotected sexual intercourse, transfusion of

contaminated blood, sharing of contaminated needles and between a mother and her baby during pregnancy, childbirth and breastfeeding.

HIV-1 transmission in breastmilk

Breastfeeding can increase the risk of HIV-1 transmission. But lack of breastfeeding, however, can expose children to an increased risk of malnutrition (poor nutrition) or infectious diseases other than HIV. All HIV-infected mothers should receive counselling that includes information about the risks and benefits of various infant feeding options, and guidance in selecting the most suitable option for their situation. The World Health Organization recommends breastfeeding for all HIV infected mothers on antiretroviral drugs (ARVs)who are virally suppressed.

What is this research study about?

The **PedMAb1** study aims to find new medicines to prevent HIV transmission from mother-to-child during breastfeeding. This new category of medicines are antibodies that neutralize the virus before it enters a cell. In **PedMAb1** we are working with **2 study products**, which are antibodies (**VRC07-523LS and CAP256V2LS**). **To make it easier for your reading we will call them VRC07 and CAP256 from now onwards in this document.** These antibodies have been already tested with success among adults. VRC07 has been tested in babies and there have been no safety concerns. CAP256 has not been tested in babies / children yet. However, there are no safety concerns in adults. The combination of CAP256 and VRC07 has not yet been tested in babies/children yet. The two antibodies can be used individually or in combination in prevention studies in babies. These drugs will work like an immunisation. We know that sometimes mothers forget to take their medicines or babies do not take their medicines or spit it out. For these reasons, we are trying to find new medicines to prevent HIV transmission from mother-to-baby during breastfeeding. In this study we will enrol babies who are born to mothers with HIV infection, but the baby tests HIV negative. Please note that the medicines / study products / antibodies used in this study are not necessarily the ones that will be produced at a large scale in factories for general use.

Study medication/ Study products / Study antibodies

Antibodies can protect from infection or disease. This strategy has been successfully used to prevent disease caused by other viral infections in children. Babies born to women with hepatitis B who receive a single dose of hepatitis B immune globulin (HBIG) are protected from hepatitis B infection. Babies at risk of serious respiratory syncytial virus (RSV) may receive monthly doses of a monoclonal antibody to protect them. There is increasing evidence that antibody, in particular neutralizing antibody, has the potential to prevent infection with HIV.

Two antibodies will be tested in the study: CAP256 was isolated in South Africa and it shows strong protection among South African viral strains in the laboratory. VRC07 also shows protection in the

laboratory and it covers more strains (types) of HIV.

Study information

The study is being conducted in South Africa only (RK Khan Hospital, Durban and surrounding delivery facilities). The recruitment period is planned for 6 months. We aim to recruit about 48 babies. Your participation in the study will last approximately 6-9 months depending on which arm (group) of the study your baby is allocated to. There are 6 arms (groups).

Expected outcome

This study will provide information on the safety and levels of VRC07 and CAP256 in babies, paving the way to efficacy studies. These results will allow us to do larger studies to measure whether these antibodies prevent HIV transmission from mother-to-baby.

Why have you been invited to participate?
You have been invited to participate in this study because you are living with HIV and you intend to breastfeed your baby.

What will your responsibilities be during this study?
For this study, you and your baby will have 9 or 16 study visits during 6 or 9 months of follow-up, depending in which study arm your baby will be randomly allocated to. The visits are planned at Days 1, 3, 14, 28 after delivery and then monthly until 6 months. If your baby receives a second dose then we will need to see you and your baby at Days 1, 3, 14, 28 after the second dose and then monthly until 6 months after the second dose.

The visits will consist of:

- Questionnaires about your child´s general health and measurement of your child's height and weight. Please bring your child´s health card(s) and your diary for us to review.
- Subcutaneous (under the skin) injections of the study products (VRC07 and CAP256) within 96 hours of birth. If you continue to breastfeed, a second dose (injection under the skin) is planned after 3 months. Your child will be randomly allocated into one of different dosing arms of the study medication. At the beginning of the study we will start with arm 1 using one injection at a low dose. We will measure safety and blood levels. Once we know how this dose is tolerated by babies in arm 1, we will move on to higher doses that are thought to be safe in babies and increase to 2 injections (both antibodies) at 2 time points. This will happen in a sequence from arms 2 until arm 6. We aim to include 8 babies in each arm.
- The number of injections your baby receives will depend on your baby's weight and age:
- **Arms 1-5:** Babies will only receive an injection at birth. They will receive 1 injection except if they fall under Arm 5 and are the maximum weight of 4kg. The 4kg baby will need 1.2mL which will be given as 2 injections.
- **Arm 6/6b:** Babies will receive 2-3 injections at birth (CAP: 1 injection of 0.6mL and VRC: 1-2 injection of 0.9mL). The second administration will be given at month 3 by 2 injections (CAP: 1 injection of 1.2mL and VRC: 1 injection

of 1.2mL). We will give as few injections as possible.

- A small amount of blood will also be taken from your child's arm using a needle at each **PedMAb1** study visit. This blood will be tested for the presence of HIV-1 and for a number of factors which indicate how the health of your child is.
- We may contact you by telephone or by home visit, if you miss any of the visits to check how you and your baby are.

Will you benefit from taking part in this study?
The results of the blood tests and medical check-ups will be discussed with you. The study staff member will also inform you of any new findings which may arise.

Are there any risks involved in your participation to this trial?
There are no anticipated serious risks to the children involved in this study. Children may experience slight discomfort, pain or bruising during blood sampling and during the subcutaneous injection. However, all attempts will be made to minimise this. The researcher is trained in counselling and will be in a position to assist you with possible discomfort regarding the testing or results of the tests and any emotional discomfort caused by the interviews.

If you do not agree to take part, what alternatives do you have?
Participation in this study is entirely voluntary. You do not have to take part in this **PedMAb1** study if you do not wish to do so. You may also stop participating in the research at any time you choose. It is your choice and all of your rights will still be respected. Refusing to participate will not affect your child care in any way. Your baby will receive the usual standard of care treatment, oral antiretroviral prophylaxis, regardless of study participation. At the present moment, apart from mother's ARV medicines and baby's oral ARV prophylaxis there are no other standard of care medicines to protect babies from acquiring HIV-1 after birth.

Who will have access to your medical records?
Any personal information collected during the study will be linked to a code and will be kept confidential. Any report derived from the study will not be linked to any personal information from the participants and will be kept confidential at all times. Auditors will review the records to ensure that all ethical and protocol conditions have been applied.

Part of the biological samples will be sent for analysis to Europe as some tests may not be available in the country of collection. If you have any questions, please do not hesitate to ask your contact person.

Any leftover of the samples collected during the study may be used in future research studies, including genetic studies.

What will happen in the unlikely event of some form of injury occurring as a direct result of your taking part in this research study?
This study has been approved by the Committee for Research on Human Subjects (The execution of the research will

comply with the World Medical Association Recommendations Guiding Biomedical Research Involving Human Subjects – Helsinki declaration).
In case of a problem, you should report it to your doctor. As sponsor, the SAMRC has taken an insurance policy which guarantees compensation if you experience any harm as a direct result of your involvement in this trial.

Please note that in accordance with the provisions of the Protection of Personal Information Act 4 of 2013 (as amended), by taking part in this research you consent to:

a. Your personal information (hereinafter 'data') being collected, processed, shared and stored in accordance with the research protocol as approved by the South African Medical Research Council's Human Research Ethics Committee (SAMRC HREC);

b. Your anonymised data being shared, processed and transferred by third parties and between third parties, and where relevant beyond the jurisdictional borders of South Africa;

c. All findings and results flowing from my anonymised data being broadly shared and published on the conclusion of the research.

Reimbursement
We will cover costs of your time, inconvenience and travel and you will be reimbursed upon completion of each **PedMAb1** study visit. The amount for a full study visit will be R450.00.

Is there anything else that you should know or do?

For more information you may contact:
Prof. Ameena Goga:
Ameena.Goga@mrc.ac.za. / Dr Logashvari Naidoo +27 31 242 3688.
Logashvari.Naidoo@mrc.ac.za

If you have any additional queries, please contact the Committee for Research on Human Subjects at the South African Medical Research Council SAMRC HREC: Adri Labuschagne, tel. (021) 938 0687; adri.labuschagne@mrc.ac.za

For this Study, you will be under the care of the site Principal Investigator (PI).

If you have not been provided with answers to your satisfaction, you should write to the South African Health Products Regulatory Authority (SAHPRA) who provides regulatory approval for the study at:

The Chief Executive Officer
South African Health Products Regulatory Authority
Loftus Park
Building A
402 Kirkness Street
Arcadia, Pretoria
0083
E-mail: Boitumelo.Semete@sahpra.org.za
Tel: 012 501 0413

- **You should inform your family practitioner or usual doctor that you are taking part in a research study.**
- **You should also inform your medical insurance company, if you have one, or your routine public health services that you are participating in a research study. We will also write this on your baby's Road to Health Booklet.**
- **You will be offered a copy of the detailed information and consent form for your own records.**
