# Supplementary material for: Safety and pharmacokinetics of subcutaneous administration of broadly neutralizing anti-HIV-1 monoclonal antibodies (bNAbs), given to HIV-1 exposed, uninfected neonates and infants: study protocol for a phase I trial
Source: BMC Infect Dis. 2024 Jul 20;24:712. doi: 10.1186/s12879-024-09588-3 (PMC11264722; doi:10.1186/s12879-024-09588-3)
Supplement: Supplementary file 4 — Supplementary Material 4: Appendix 4.word Table 7: Schedule of sample collection for HIV exposed infants without HIV: ARMS 1 to 5. And Table 8: Schedule of sample collection for HIV exposed infants without HIV: ARMS 6/6b. Schedule for collection of the biological samples of arms 1-5 and arms 6/6b. [file 12879_2024_9588_MOESM4_ESM.docx]

**Appendix 4**

The schedule for collection of the biological samples of arms 1-5 and arms 6/6b are displayed in the following Tables.

**Table 7:** Schedule of sample collection for HIV exposed infants without HIV: ARMS 1 to 5.

|  | | | | | **Within 96 h** | | **DAYS**  **post- bNAb administration** | | | **MONTHS**  **post- bNAb administration** | | | | |
| --- | --- | --- | --- | --- | --- | --- | --- | --- | --- | --- | --- | --- | --- | --- |
|  | | | | | **Screening** | **Enrolment** | **3** | **14** | **28** | **2** | **3** | **4** | **5** | **6 - EOT** |
| **Sample Type** | **Tube type and assay** | **Tube/ Swab/**  **Sample number collected** | **Total volume to collect**  **(mL)** | **Assay**  **Location** |  |  |  |  |  |  |  |  |  |  |
| **Whole Blood** | **EDTA**  **for POC HIV-1 Qualitative Test and DBS for PK#** | **1 x 0.5 mL** | **0.2 mL** | **CRSL** | **X** | **-** | **X** | **X** | **X** | **X** | **X** | **X** | **X** | **X** |
|  |  |  | **70µL blood per DBS up to 0.4mL** | **CRSL then research lab** | **-** | **X** | **X** | **X** | **X** | **X** | **X** | **X** | **X** | **X** |
|  | **EDTA**  **Hematology** | **1 x 0.5 mL** | **0.5 mL** | **BARC** | **X** | **-** | **-** | **-** | **X** | **-** | **-** | **-** | **-** | **-** |
|  | **HCT** | **Heel prick/**  **Venous Blood** | **One drop** | **Hospital/**  **CRS** | **-** | **X** | **X** | **X** | **X** | **X** | **X** | **X** | **X** | **X** |
| **Serum** | **SST**  **Chemistry** | **1x 0.5 mL** | **0.5 mL** | **BARC** | **X** | **-** | **-** | **-** | **X** | **-** | **-** | **-** | **-** | **-** |
| **Serum** | **SST**  **neutralising activity,**  **anti-bNAb Abs,**  **PK*** | **3 x 0.6 mL** | **1.8 mL** | **CRSL then research lab** | **-** | **X** | **-** | **-** | **X** | **-** | **X** | **-** | **-** | **X** |
|  | **SST**  **neutralising**  **activity** | **1 x 0.6mL** | **0.6 mL** |  | **-** | **-** | **-** | **-** | **-** | **-** | **-** | **X** | **-** | **-** |
| **Oral Fluid** | **N/A**  **PK - ELISA** | **1 x Swab** | **N/A** | **CRSL then research lab** | **-** | **X** | **X** | **X** | **X** | **X** | **X** | **X** | **X** | **X** |
| **Total volume of blood** | | | | | **1.2 mL** | **2.1mL** | **0.5mL** | **0.5mL** | **3.3mL** | **0.5mL** | **2.3mL** | **1.1mL** | **0.5mL** | **2.3mL** |
| **Allowable volume as per initial submitted protocol version 1.0** | | | | | **1.2 mL** | **2.4 mL** | **0.6 mL** | **0.6 mL** | **3.6 mL** | **0.6 mL** | **2.6 mL** | **1.6 mL** | **0.6 mL** | **2.6 mL** |

# At screening, only 0.2 ml blood will be drawn for HIV diagnosis (POC test). At enrolment only 0.4mL blood will be drawn for Dried Blood Spots (DBS) for PK. At all other timepoints the 0.5mL EDTA blood will be used for HIV diagnosis POC test (0.1mL), and a repeat test if needed (total 0.2mL). The remaining blood will be used to spot the Cards with 70µL for each spot. *This blood will be used to double check / validate the DBS PK, intermittently or as appropriate / needed.

**Table 8**: Schedule of sample collection for HIV exposed infants without HIV: ARMS 6/6b.

|  | | | | | **Screening**  **Within 96 h** |  | **DAYS post – administration of 1^st^ bNAb** | | | | | **DAYS post- administration of 2^nd^ bNAb at 3 months** | | | | **MONTHS**  **post- administration of 2^nd^ bNAb**  **at 3 months** | | | | |
| --- | --- | --- | --- | --- | --- | --- | --- | --- | --- | --- | --- | --- | --- | --- | --- | --- | --- | --- | --- | --- |
|  |  |  |  |  |  | **Entry/**  **Enrolment Within 96 h** | **1** | **3** | **14** | **28** | **90** | **1** | **3** | **14** | **28** | **2** | **3** | **4** | **5** | **6-EOT** |
| **Sample Type** | **Tube type and assay** | **Tube/ Swab/ Sample number collected** | **Total volume to collect (Ml)** | **Assay Location** |  |  |  |  |  |  |  |  |  |  |  |  |  |  |  |  |
| **Whole Blood** | **EDTA**  **For POC HIV-1 Qualitative Test**  **and DBS for PK#** | **1 x 0.5 mL** | **0.2 mL** | **CRSL** | **X** | **-** | **-** | **X** | **X** | **X** | **X** | **-** | **X** | **X** | **X** | **X** | **X** | **X** | **X** | **X** |
|  |  |  | **70µL blood per DBS up to 0.4mL** | **CRSL then research lab** | **-** | **X** | **X** | **X** | **X** | **X** | **X** | **X** | **X** | **X** | **X** | **X** | **X** | **X** | **X** | **X** |
|  | **EDTA**  **Hematology** | **1 x 0.5 mL** | **0.5 mL** | **BARC** | **X** | **-** | **-** | **-** | **-** | **X** | **X** | **-** | **-** | **-** | **X** | **-** | **-** | **-** | **-** | **-** |
|  | **HCT** | **Heel prick/ Venous Blood** | **One drop** | **Hospital/**  **CRS** | **-** | **X** | **X** | **X** | **X** | **X** | **X** | **X** | **X** | **X** | **X** | **X** | **X** | **X** | **X** | **X** |
| **Serum** | **SST**  **Chemistry** | **1x 0.5 mL** | **0.5 mL** | **BARC** | **X** | **-** | **-** | **-** | **-** | **X** | **X** | **-** | **-** | **-** | **X** | **-** | **-** | **-** | **-** | **-** |
| **Serum** | **SST**  **Neutralising activity,**  **Anti-bNAb Abs,**  **PK*** | **3 x 0.6 mL** | **1.8 mL** | **CRSL then research lab** | **-** | **X** | **-** | **-** | **-** | **X** | **X** | **-** | **-** | **-** | **-** | **-** | **-** | **X** | **-** | **X** |
|  | **SST**  **Neutralising**  **Activity, pK*** | **1 x 0.6 mL** | **0.6 mL** | **CRSL then research lab** | **-** | **-** | **X** | **-** | **-** | **-** | **-** | **X** | **-** | **-** | **X** | **-** | **X** | **-** | **-** | **-** |
| **Oral Fluid** | **N/A**  **PK – ELISA** | **1 x Swab** | **N/A** | **CRSL then research lab** | **-** | **X** | **-** | **X** | **X** | **X** | **X** | **-** | **X** | **X** | **X** | **X** | **X** | **X** | **X** | **X** |
| **Total volume of blood** | | | | | **1.2mL** | **2.2mL** | **1mL** | **0.5mL** | **0.5mL** | **3.3mL** | **3.3mL** | **1.1mL** | **0.5mL** | **0.5mL** | **2.1mL** | **0.5mL** | **0.5mL** | **2.3mL** | **0.5mL** | **2.3mL** |
| **Allowable volume as per initial submitted protocol version 1.0** | | | | | **1.2mL** | **2.4 mL** | **0 mL** | **0.6 mL** | **0.6mL** | **3.6 mL** | **3.6 mL** | **0 mL** | **0.6mL** | **0.6mL** | **2.6mL** | **1.6 mL** | **0.6mL** | **2.6mL** | **0.6mL** | **2.6 mL** |

# At screening, only 0.2 ml blood will be drawn for HIV diagnosis (POC test). At enrolment and day 1 after bNab administration only 0.4 mL blood will be drawn for DBS for PK. At all other timepoints this 0.5mL EDTA blood will be used for HIV diagnosis POC test (0.1mL), and a repeat test if needed (total 0.2mL). The remaining blood will be used to spot the cards with 70μL for each spot.

*This blood will be used to double check / validate the DBS PK, intermittently or as appropriate / needed.

Safety tests include: Haematology: Full blood count (FBC) with differential (diff) + platelets; and Chemistry: Alanine Transaminase (ALT), Aspartate Aminotransferase (AST), Total Bilirubin, Creatinine
